# Abstracts of papers presented at the ISLAR (International Symposium on Laboratory Automation and Robotics) 2000

**DOI:** 10.1155/S1463924601000013

**Published:** 2001


Journal of Automated Methods & Management in Chemistry, Vol. 23, No. 1 (January–February 2001) pp. 1–37

### Abstracts of papers presented at the ISLAR (International Symposium on Laboratory Automation and Robotics) 2000

The 18th International Symposium on Laboratory Automation and Robotics provided presentations on state-of-the-art developments in laboratory automation and robotics. The symposium programme included papers and posters on all aspects of the technology. These comprised: managing laboratory automation (drug discovery); bioanalytical analysis; managing laboratory automation in drug discovery development and QC laboratory; functional genomics strategies and high throughput screening; advanced integration strategies; method development and global methods transfer; compound handling and logistics; combinatorial chemistry and automated synthesis; high throughput LC-MS-MS; increasing efficiency in dissolution testing; lead optimization; strategies for UHTS; increasing throughput for ADME toxicology; data management/data handling and bioinformatics; using contract laboratories to increase productivity; assay miniaturization; process optimization; compliance and automation—the regulatory perspective; novel high throughput screening technologies; compliance and automation—the industry perspective. Several discussion sessions were included and activated, and provided interactive communication on a wider range of subjects.

Although the programme was very comprehensive, the Symposium was designed to provide time for both formal and informal exchange of information. The technical presentations were organized into concurrent sessions with grouped papers on related topics.

Abstracts for each paper and each poster are included here. Full presentations of several of these papers will appear in later editions of this journal.

### Genomics and the new era of drug discovery: a Wall Street perspective

*E. James Streator, III, Director of Healthcare Investment Banking and Partner, Thomas Weisel Partners, NY*

The new era of drug discovery is revolutionary, it is complicated and it is just beginning. Wall Street's enthusiasm for and lack of understanding of industry participants has been demonstrated by its investment history. In the last twelve months, investors bid genomics and drug discovery stocks up an average of 400%, then proceeded to cut valuations in half, only to bid prices up again.

As a whole, genomics companies trade at a premium to the tools companies at approximately 30× 2001 revenue estimates. We believe Wall Street grants genomics companies a premium because it perceives them as owning the data, and thus having a tie to the enormous potential upside. We expect that revenue growth, new technology breakthroughs, and new biological discoveries will drive valuations for these companies over the foreseeable future.

Drug discovery tools and instruments companies trade at a wide range of multiples, from microarray companies trading at 40× 2001 revenue to consumables and equipment companies trading at 15–20×. As 'picks and shovels' infrastructure plays, investors look for revenue visibility and recurring revenue. In addition, the market does not fully appreciate the relationship between instruments and related technology and Moore's law (the doubling of processing speeds every 18 months). We expect valuations to be driven by revenue growth and collaborations for the foreseeable future.

### Productivity and drug discovery: the Genzyme small molecule experience

*Frederik Vinick, Senior Vice President of Drug Discovery, Genzyme Corporation, Cambridge, MA*

Drug Discovery has always been a challenging, difficult, expensive proposition, appropriately characterized as high risk/high return. The pharmaceutical industry has a distinctly difficult time generating its product, new drugs, in a timely, predictable, cost-effective manner. Many factors, a good number of them intrinsic to the complex science of novel drug discovery, contribute to this problem. As the industry has moved from the era of 'me, too' imitative products to the current age of genomics, new targets and a heavy emphasis on poorly treated diseases, the challenge at hand (and the attendant risk and uncertainty) has become even more severe.

Thus, at a time when we have access to many powerful new technologies which, in principle, offer us new, more effective solutions to the daunting problems we must solve, it is worthwhile, in fact necessary, for us to consider, prudently and thoughtfully, the best uses of these tools. Automation, miniturization, surrogate *in vitro* models, less *in vivo* testing—all of these notions are at face value virtually unquestionable, incontrovertible major steps in the right direction towards more productive drug discovery. However, we have to guard against the use of these clever tools in ways that actually create more work and cost.

In this talk, we describe the Genzyme approach to drug discovery, a hybrid of methods, new and old, which seeks to minimize risk/cost and maximize the output of drug candidates (novel substances which are safe and efficacious in animal models of disease). We will illustrate our strategy with data from several actual examples. We will also propose a research process which we believe illustrates a highly efficient use of modern technologies.

### Modern HTS: infrastructure and workflow versus technology and science

*Melvin Reichman, Dupont Pharmaceuticals, Wilmington, DE*

The advances in HTS technologies over the past decade have been impressive; however, raw HTS remains a minor component (<3%, by time) of the typical Discovery project life-cycle. If we postulate the view that drug screening in Pharma represents a manufacturing process wherein the deliverables are *bona fide* development candidates, rate-limited steps can be proactively identified and mitigated. The talk will review by example the role of project management in the overarching disciplines comprising the extended drug screening process. Centralized HTS, compound handling, quality control and assurance and information management are presented as core components of the modern drug discovery infrastructure that enable the efficient execution of hit-to-lead operations through to drug Development.

### Accelerated lead identification: a result of process industrialization

*Berta Strulovici, Merck & Co., North Wales, PA*

The industrialized era in pharmaceutical discovery will require creativity and innovation in each component of the drug discovery machinery. Three years ago, Merck Research Laboratory formed a dedicated HTS unit, to screen organic compound and natural product libraries for new therapeutic lead candidates. This is a stand-alone facility that integrates assay development, compound management, ultra-fast robotics, screen miniturization technologies, and complex data management systems linked by computerized control networks. Our approach to HTS is based on intact cell and biochemical systems using the power of robotics capable to operate 24 hours/day, for weeks at a time. I will present specific examples where our approach of combining novel assay technologies with innovations in automation and software applications proved fruitful, as attested by the identification of lead candidates in several new therapeutic areas in record time.

### Fostering innovation in the corporate laboratory

*John Babiak, Pharmacopeia, Princeton, NJ*

Opportunities for technological innovation in the lab continue to appear at a rapid pace. Screens miniturized to as small as 1 µl have been performed on the scale of over 200,000 wells. Novel, homogeneous formats are becoming available that can simplify many assay types. Automation and scheduling software continue to improve both speed and flexibility of screening systems. At a superficial level it appears that all things are possible with sufficient resources and time to implement the correct combination of technologies. One question to consider, therefore, is what should the process be to support innovations within the corporate laboratory. No laboratory can evaluate and implement every technical tool that is available. Development of new technologies in the corporate lab setting, such as in high throughout screening (HTS), requires the balancing of multiple factors:

- Productivity versus innovation
- Established products versus high-risk beta (or alpha) testing
- Buy versus build versus co-develop
- Big solutions versus small solutions
- Focus on a few specialties versus create a broad portfolio of capabilities
- Involvement of staff in technology development versus screening

All these issues are influenced by the corporate environment (and goals), staffing, resources and history. Therefore, it is important to create an atmosphere where novel ideas are invited and discussed, some are explored, the most valuable are implemented and mistakes are educational.

### Managing the evolution of screening: from decentralized HTS to centralized UHTS

*Mary Jo Wildey, The R. W. Johnson Pharmaceutical Research Institute, Raritan, NJ*

The Drug Discovery lead identification process has been evolutionary, with goals directed toward shortening the time needed to develop new chemical entities. Traditionally, many organizations have chosen decentralized approaches to screening. This approach utilizes personnel resources from the research teams to perform the screens and resources from a central automation team to provide programming, validation, and data processing support. Due to hardware and assay format technology advances, many organizations have experienced significant increases in throughput capabilities. In many cases, these increased capabilities are the basis for the change to centralized screening groups where the automation team performs the bulk of the screening operation with limited assistance from the research teams.

We shall discuss the practical aspects of making the change from decentralized to centralized screening platforms. Topics including hardware and software impacts, system support, and impact on personnel will be addressed. We will also discuss ideas on diminishing the risks that accompany technology shifts and the practical management of this process.

### Semi-automated sample preparation 96-well format extractions

*Anita Shen, Huong Mai, Min Chang, Hope Skriba, Qin Ji, Ray Wieboldt, Daniel Daszkowski and Tawakol El-Shourbagy, Abbott Labs, Abbott Park, IL*

Sample preparation has always been a time and labor consuming and error-prone step in the workflow of a bioanalytical laboratory. Since the development of user friendly LC/MS and its acceptance as a HPLC detector, sample preparation has become the bottleneck of the whole process. In order to improve the quality and reduce the labor required to perform a traditional manual solid phase extraction and liquid-liquid extraction, automated instruments were introduced. The first generation laboratory robots such as Zymark Zymate, Perkin-Elmer MasterLab and Waters MilliLab are serial instruments. Although advanced users may program some first generation robots to do several activities in parallel, most of the activities have been performed in series. As a result of this serial nature, the throughput of the robotic assay was about 6 to 12 minutes per sample. To increase the throughput, solid phase extractions were performed using individual workstation or workstation banks, then the robotic arm was freed up to perform other tasks. Another approach is to perform the steps in large array formats such as the 96-well microtitre plate.

In this presentation, the *Hamilton* **Micro Lab AT** was used as a re-formatting workstation, which transfers the plasma samples from clinical tubes to 96-well plates. The Micro Lab also added the internal standard, treatment reagents for solid phase extraction (SPE) and organic solvents for liquid-liquid extraction (LLE). The advantage of the Hamilton is its ability to pipette 12 samples in each cycle reliably. For LLE, the 96 well plate was sealed with a polyethylene film by heat (**Comb sealer** from *Marsh Biomedical*). Extraction was performed by shaking, centrifuging and finally, phase separation with the *Hamilton* **Micro Lab**. For SPE, the treated sample in 96-well plates was transferred to SPE workstation such as *Beckman* **Biomek 2000 or** *Tom Tech* **Quadra** for further processing. Using those workstations, an analyst was able to extract four 96-well plates and perform the LC/MS analysis in one workday with high reproducibility. Several semi-automated, 96-well format LLE or SPE, LC/MS assays were validated and used to support clinical trials.

### Collecting sample weight data on various liquid handling robots

*Jimmy Bruner, Larry Birkemo, Kelly Jordan, Glenn Smith, and James Ormand, Glaxo Wellcome Inc., Research Triangle Park, NC*

Liquid-handling platforms often do not provide a mechanism for collecting weight data needed for instrument qualification and sample transfer confirmation. This paper discusses the development, implementation, and application of a system that facilitates liquid-handling confirmation required for Good Laboratory Practice (GLP) compliance and provides an avenue to track amount of sample transferred for extraction.

The Balance Data Collector (BDC) system was designed as a flexible generic balance tool to be used with Tecan Gemini© and Packard WinPrep® software. The BDC system provides a user interface for balance configuration, a pointer to a file for storing weight data, and an external interface through command line arguments.

BDC is currently used for instrument qualification and sample collection in bioanalytical applications. Instrument qualification includes refining instrument liquid classes and verifying pipetting accuracy and precision. For bioanalytical applications using 96-well plates, BDC collects individual aliquot weights of samples transferred during an assay.

The BDC system gives us the capability to control a balance via liquid-handling programming platforms such as Tecan Genini© and Packard WinPrep®. Integration of liquid-handling platforms and BDC reduces the time the scientist must spend recording weight data needed for GLP compliance and can be used to increase accuracy of calculated sample concentrations.

### Laboratory automation strategies for method development and sample analysis

*Roger Coe, JD Hulse and JW Lee, MDS Harris Laboratories, Inc.*

**Purpose**. The routine use of LC/MS/MS has shifted the rate-limiting step for high-throughput sample analysis from instrument to sample processing. Solid-phase extraction (SPE) in the 96-well format allows fast sample extraction from biological matrices. Our purpose was to work out a strategy exploiting the advantages of two different types of SPE automation: The flexible programming of Zymark RapidTrace® was used for effective method development, and the method is then adapted to Tomtec Quadra-96® for high sample throughput. Additional automation such as liquid-handling robots (Tecan Genesis RSP) and barcode reading supplements the 96-well assay for fast sample turnaround.

**Methods**. The RapidTrace® workstation was programmed to conduct multiple experiments to screen SPE sorbents for the target analytes in non-matrix solutions. Conditions for the desirable interaction of the analytes with the sorbent were investigated and selected. The SPE rinsing and eluting parameters were tested using different combinations of solvents to wash unwanted components away and optimally recover the analytes from the biological matrix. The chosen method was adapted to 96-well on the Quadra-96®, which allows all 96 samples to be processed simultaneously. Previous validated methods with SPE cartridges were also optimised using the RapidTrace® and the methods transferred to the 96-well format on the Quadra 96®.

**Results**. The outlined strategy was applied successfully to develop assays for several compounds in a short period of time. Examples are provided for a synthetic narcotic, basic drugs, and progestins. Previous cartridge SPE methods of tamoxifen and $\Delta^9$-tetrahydrocannabinol and its hydroxylated metabolite were transferred to the 96-format. The typical method development time was less than 5 days. Typical sample processing time for 96 samples was 15 to 50 min. Inter-plate variability was tested to extend from single to multiple plates within a batch run. When samples were aliquoted by the Tecan Genesis®, at least six plates could be extracted in one day.

**Conclusion**. A rational and efficient approach for method development and validation using two different automated SPE systems was effective in providing fast turnaround time to support the fast paced LC/MS instrument.

### 384-well solid phase extraction: strategies and limitations

*Geoffrey Rule, Advanced BioAnalytical Services, Ithaca, NY*

Solid-phase extraction (SPE) has been widely adopted as a sample preparation technique since its introduction in the 1970s. Along with liquid-liquid extraction and 'on-line' sample preparation techniques it fills a substantial portion of the sample preparation needs of high-throughput laboratories. Since the introduction of 96-well SPE over four years ago, more and more laboratories with high-throughput requirements have moved to this format as a means of increasing sample preparation productivity.

Recently, we have demonstrated SPE in a higher density, 384-well format for the drug methotrexate and its 7-hydroxy metabolite in both human urine and plasma. The method makes use of 5 µm C18 particles entrapped within a glass fiber disk and a semi-automated robotic workstation. The ability to prepare and analyze 384 samples as a single analytical run has been demonstrated with good precision and accuracy. A second method, previously developed and validated in 96-well SPE format for an undisclosed drug, has also been modified for a 384-well format cross-validation. The results of these investigations will be presented along with discussion on the practical aspects (general strategy, instrumentation, and limitations) of 384-well sample preparation and its potential for increasing instrument utilization and reducing data turnaround times.

### New century...new applications for automation in pharmaceutical analysis

*Stephen Scypinski, Ronnie McDowell, Ron Muenz, John Troisi, Adam Fermier, Analytical Chemistry Research & Development, The R. W. Johnson Pharmaceutical Research Institute, Raritan, NJ*

The advent of the new millennium brings new challenges and opportunities for automation in the pharmaceutical analysis laboratory. While utilization of workstations for automated assay, content uniformity and dissolution in the analytical laboratory continues to dominate the landscape of pharmaceutical analytical chemistry, other automation arenas are beginning to emerge.

The rapid expansion of drug discovery efforts in most pharmaceutical companies, coupled with the utilization of high throughput screening and combinatorial chemistry has increased the demand for analytical data at a very early stage in the drug development process. The desire to choose the proper development candidate is based not only on preclinical data but also physiochemical information. The large amount of compounds that must be screened for such characteristics as pH/solubility profile, octanol-water partition coefficient and other preformulation parameters generates a great deal of analytical work that must be performed. To complicate matters, one is generally sample-limited at this phase of development.

We have begun to explore options for automation of such early phase preformulation activities in our laboratory to provide better and more timely support for our customers. By utilizing the flexibility of general laboratory automation and the ability of drug discovery to work with small quantities of sample, we have constructed systems that perform analyses and measurements for early drug development candidate screening. In addition to conventional laboratory automation, we have also explored the route of 'parallel processing' and miniaturization as another means of obtaining more data on a limited amount of sample more quickly. Examples of the various systems constructed and used in our laboratory and their impact on drug development will be discussed.

### Learnings for introducing automated dissolution, automated HPLC & new technology

*Jay Makwana, Boots Healthcare, Nottingham, UK*

Having introduced new technology into drug product development laboratories to deal with demands for increasing workload and improvements in pace of analytical work, I will share our learnings. In this presentation, I will cover the introduction of automated dissolution, automated HPLC, capillary electrophoresis and a networked data handling system.

I will also reveal some accounts of the softer challenges of getting buy-in from the staff, converting high scepticism to love in some cases. Other key steps of evaluation, preparing purchase proposals, implementation planning, validation, resource selection and importance of enthusiastic champions, continuous improvement and project management will also be addressed. A flow chart of a recommended process is one of the key outcomes to help with introduction of new technology and passionately pursue success.

### A practical strategy for utilizing standard automation technology across multiple components

*Michael Rutherford, Eli Lilly and Company, Lilly Corporate Center, Indianapolis, IN*

As the pharmaceutical industry continues to change, more emphasis is being placed on the identification and development of new and innovative compounds and products. To support the rapid development and increased number of these new chemical entities (NCE's), utilization of laboratory automation in the product development and launch process is becoming increasingly important to support 'Speed to Market' initiatives. While utilizing laboratory automation can provide significant benefits, it is also important to consider standardizing that technology. Standardization of automation technology and platforms across the various components involved in the process can be very beneficial by providing increased capacity, better resource utilization, improved quality of results across the organization, detailed documentation and audit trials, and easier method transfer.

Over the last decade, laboratory automation has been utilized effectively by Analytical Development and Quality Control at numerous sites throughout Eli Lilly and Company through the implementation of a variety of automated solutions and technology to address specific needs. Many of these systems were developed 'in-house' and/or were custom applications, resulting in some replication and standardization across various components. However, as many of these solutions have become older and support resources have changed, more emphasis is being placed on purchasing 'off-the-shelf' solutions. As these automated systems are being replaced, standardization of automation technology and platforms are also being considered. To initiate this strategy, Analytical Development and Quality Control in Indianapolis have begun to develop and implement several standard automated solutions across their organizations with initial emphasis on solid dosage form testing. The strategy, technology, benefits, learning points and experiences, current project status, and future plans will be discussed during this presentation.

### Delivering a highly automated drug product laboratory—the importance of system integration standards

*Perry Hailey, Pfizer Central Research, Sandwich, UK*

### Identification of cytokines and hormones inducible anti-apoptotoic genes by microarrays

*Grace Wong, Serono Reproductive Biology Institute, Randolph, MA*

### Design and development of a novel arraying machine

*Steven Frosdick, Radius Biosciences, Medfield, MA*

Microarrays consist of spots of material aligned on a regular grid pattern. Typically these spots have a diameter of ~200–700 μm and are placed onto flat substrates at a spot-spot distance of 2–3× the spot diameter. Such arrays of spots have increasing applications in the drug discovery effort. For example, spots of DNA can be used to determine the modulation of genes in diseased vs normal tissue, or arrays of chemical compounds can be used to screen for interactions between these moieties and receptors or enzyme targets.

A machine will be described that allows for arrays to be generated on a variety of substrates. The latter can include glass slides or nylon membranes. The novel design (patent pending) allows for a high substrate capacity while maintaining a footprint small enough to fit on a lab bench. Other features which allow for the rapid generation of arrays will be discussed as well as features which allow for more consistent spot morphology. The machine has a molecular design to allow it to be used as a personal spotter or upgraded as a high volume production machine. This common platform ensures reduced cost without compromising performance. Intuitive software enables arrays to be made from either 96-well of 384-well source plates. The importance of these design features will be discussed.

### Process management software for MicroArray gridding

*Frances Stewart, C. Teall, B. Gillespie, SmithKline Beecham Pharmaceuticals*

Use of MicroArrays for examination of differential gene expression across tissues and treatments has dramatically increased over the last 2 years. Increasing data volumes have created a bottleneck in the process, namely, sample tracking and data management. Development of a fully functional process management software system posed several challenges to informatics groups at SB. Fundamental requirements included:

- Integration with evolving clone and tissue registration databases
- Maintenance of synchronous data sources involving multiple validations of data integrity
- Integration with internal and external image analysis software
- Integration with internal and external data quality software
- Integration with multiple robotics platforms
- Tracking plate lineage necessitated by clone and control sample re-positioning within slides
- Ability to easily incorporate or change multiple plates to slide mapping schemes for spotting

The SB Cheminformatics group developed a process management application, MAGNET, to track samples and data for gridding experiments that provided the functionality above for open dimensional plates and slides. Analyzed and normalized expression data published to our proprietary gene expression database are linked to experimental ancestry attributes tracked in MAGNET. The architecture and software tools used to build our system are discussed and a short demo of current functionality will be included.

### High throughput functional genomics

*Henry Long, Aventis Pharmaceuticals, Cambridge, MA*

With massive advances in genomics technology in the past few years, genes are being discovered far faster than the functions of the proteins for which they encode can be characterized. This is rapidly becoming a key bottleneck in target identification. We will discuss how automation technologies assist Aventis scientists in addressing these concerns. High throughput DNA purification is necessary for many processes and vendors have been slow to develop 'genomics scale', low cost solutions. A novel magnetic bead based approach will be described that fully automates DNA minipreps; it has been in production at the Cambridge Genomics Center since January 2000. A significant hindrance to functional studies is the time-consuming restriction enzyme digest and ligation process required for transferring a target gene into each different expression vector. Determining gene function can require numerous functional studies, each using a different expression vector, also optimizing protein production can require trying multiple vectors. Recently, several cloning systems have appeared on the market that substantially reduce the amount of effort involved, and, even more importantly, they are highly amenable to automation. We will describe our experiences with the Gateway$^{TM}$ System from Life Technologies and the automation involved in setting up a high throughput pipeline.

### Maximizing the benefits of automation technologies in the world of high throughput screening

*Barbara Hynd, Proctor and Gamble Pharmaceutics, Cincinnati, OH*

The field of throughput screening would not exist as we know it without the benefits provided by automation. The challenge to all practitioners of the science is how to utilize the technology in such a way as to extract the maximum benefit from existing equipment and processes without compromising speed and accuracy, or falling behind in the continuing technological advance. Maintaining this balance as well as the balance between innovation and ‘manufacturing’ processes is challenging for all HTS groups and is becoming more so in the current climate of speed, reducing costs and scarcity of trained personnel.

During this presentation some of the creative uses of automation technology will be outlined using practical examples of the differing approaches taken by individual screening groups.

### Utilization of peptides as surrogate ligands in high throughput screening

*Dale Christensen, Novalon Pharmaceutical Corp., Durham, NC*

Peptides that bind with high affinity to drug targets can be isolated from phase display libraries. If these peptides bind to the target protein at sites that are critical for biological function they could be used as surrogate ligands in a simple ligand binding-type assay for HTS. To test this hypothesis, we have performed extensive testing to determine whether the affinity selected peptides bind at biologically relevant sites on enzymes and other classes drug targets. These peptides bind to biologically relevant sites in the majority of cases tested to date and have been shown to inhibit enzyme function and critical protein:protein interactions. Further, these peptides can be used as surrogate ligands to detect small molecule inhibitors of these targets. We have utilized these peptides to develop HTS assays that can be run with luminescence, time-resolved fluorescence (TRF), fluorescence polarization (FP), fluorescence resonance energy transfer (FRET), and scintillation proximity (SPA) detection. The results of our validation data and our screening efforts will be presented.

### The difference between vintage and antiquated: the use of kinetic assays for HTS at vertex

*Mark Namchuk, Vertex, Cambridge, MA*

Kinetic assays (spectrophotometric or fluorescence) have long been the method of choice for characterization of enzymes and enzyme inhibitors. This is because kinetic assays are less susceptible to artefacts and provide more precise estimates of the rates of reaction than the equivalent end point assay. However, kinetic readouts are not widely used in HTS, mainly due to problems with protein consumption and low throughput. To address these issues we have developed a fully automated 384 assay, run on a Zymark platform fitted with a pair of Hypertask quadrant positioners and a Biorad Ultramark UV/Vis plate reader. Data obtained with the upgraded system is of equivalent quality to its 96 well predecessor and when fully operational will provide 4 x the throughput of the original system (~25,000 data point/day). Test data obtained with JNK-3 highlights the main assay development issue with 384 kinetic data, which is mixing artefacts. Now that the format is validated, it continues to provide the advantages we observed with 96 well kinetic screening.

- It is the method of choice with proteins whose substrates do not easily lend themselves to labelling.
- Very short assay development times (usually 2 weeks from receipt of protein to HTS run).
- High data quality provides excellent follow-up rates (usually >50%).
- Superior starting points for both combinatorial chemistry and modelling.

Examples of these data from Vertex programs will be highlighted.

### Use of IDE RAID level I (mirroring) for maximum data integrity on a Zymark Zymate system

*Richard Spann, Berlex Biosciences, Richmond, CA*

The Zymark System V architecture makes use of a standard IBM compatible computer (PC) for certain functions. One aspect of the PC to System V controller interface is data acquisition and storage.

Certain automated procedures require a repository for data that is generated during the course of the run. The source can be analytical instrumentation or a database at a network location. Depending on the volume of data, the floppy disk drive built into the Zymark controller may not be of sufficient capacity to serve as a target for data storage.

Because of the Windows based Zymate Utilities, any drive available from Windows can be accessed by the System V controller for writing data files. Sending across a network, however, carries the risk of an interruption in network service. The most reliable target is a hard drive installed in the interface PC. Nowadays, large capacity hard disk drives can be obtained at very reasonable prices.

To protect against data losses due to power failures, disk corruption and mechanical failure, regular backups of the desired drives are routinely performed. Unfortunately, in the event of a data loss, changes made to the faculty disk after the last backup will not be recoverable. By implementing redundancy into the data storage device, the incidence of data loss can be reduced to 0%.

The interface PC currently installed on the Zymark Zymate XP system in my laboratory was fitted with an IDE RAID level 1 (mirroring) adapter. An IDE solution was chosen because of cost. IDE hard drives are cheaper than SCSI and fast enough for the applications in use at my site. The RAID adapter will work with ANY pair of IDE drives—even of differing capacities. There is no performance hit since special software drivers are not necessary. Both master and slave disks can be mirrored from the same adapter if desired.

In combination with regularly scheduled backups of the mirrored drives, there should never be a loss of information. Since the chance of both drives in the pair failing at the same time are remote, data collection during the automated run will not be interrupted. If a problem with one of the mirrored disks develops, the adapter activates an audible alert so that the operator can install a new disk and rebuild the mirror using the good drive.

### The design of flexible workstation control software

*Claude Dufresne and Miguel Maccio, Merck Research Laboratories, Rahway, NJ*

Commercial workstations are usually controlled by a software application that was created by the manufacturer of the workstation. Depending on the features of the application, integrating the workstation into a robotic environment, or simply integrating its operation with existing databases, can be difficult. The iterative process of going back to the manufacturer for software modifications is neither satisfying nor necessarily timely.

Facing issues of robot integration, database integration, and even workstation operation, we decided to create an in-house workstation control software application. The application replaces the manufacturer-provided front-end control software altogether. The design of the software application and how it was implemented on a Bohdan Workstation will be presented.

### Personal automation: a robot on every bench

*James Gill II, Department of Research Technology, Glaxo-Wellcome, Research Triangle Park, NC and Department of Molecular Sciences, GlaxoWellcome Medicines Research Center, Stevenage, Hertfordshire, UK*

During the early 1980s computers broke out of the computer centers and onto the desktop. Today we have a spectrum of computing options from desktops for routine tasks to supercomputers for computationally intensive tasks. In a similar way, increasing familiarity with automation and the decreasing complexity of many assays have allowed robots to escape the automation suite where they are providing automation for the average drug discovery scientist. Hopefully, as with PC's, automation will develop that is scaleable so that bench top systems will handle some assays and the uHTS sytems will handle the automation intense applications.

Our group has been developing simple 'personal' robotics, which are inexpensive and flexible enough to justify wide scale distribution. In addition we feel that these small systems allow construction of dedicated robots for simple tasks, assays platforms and even portions of assays on larger robots. These systems are based on the Zymark Twister and typically consist of a Twister, a liquid dispenser and a reader. Systems have so far been developed to support measurement of solubility, melanophore screening, general enzyme screening, general receptor screening and quality control measurement. These systems have run 500K HTS campaigns successfully.

In order to insure the accessibility of the systems to the average scientist we had to insure that inexpensive and simple software was available and so we developed Scrippy. Scrippy is a multi-tiered programming environment. In one tier Scrippy presents a simple interface which enables the scientist to sequence operations between the instruments by dragging and dropping commands. However, more advanced users can write in a scripting language and develop complex commands which can then be dragged and dropped.

The increasing diversity in robot 'style' has placed a serious demand on automation support staff to provide solutions for a broad portfolio of automation systems. We have therefore tried to insure that the bench top systems are 'scalable'. We are hopeful that the systems will deal with larger numbers by being replicated. Automation intensive tasks would then be run on similar instruments and with the same software drivers on large uHTS systems thus providing a seamless automation environment.

### Practical aspects of developing automated methods for solid dosage formulations

*Alger Salt, Glaxo Wellcome Inc., Pharmaceutical Development, Research Triangle Park, NC*

Chemical analysis of pharmaceutical dosage forms is an essential part of assuring product quality. Two common analytical tests for solid dosage forms are the determination of drug content and impurities associated with a batch of product and assessment of the dosage uniformity of individual units. For chemical analysis of solid dosage formulations these tests often employ shaking, sonicating, and/or homogenizing techniques to disperse the tablet and extract the drug into a suitable solvent. In this presentation, results obtained from a set of experiments designed to evaluate the effect of energy transfer through parameters such as homogenizer speeds, sonication times, etc will be used to compare these extraction techniques. Useful tips and guidance (derived from years of experience with the Zymark TPWII tablet processing workstation) for developing and validating automated methods will also be presented.

### Enhancing the automated method technology transfer process

*Timothy Diehl, Lillian Vazquez, Angelica Chevere, Quality Assurance Department, Janssen Pharmaceutica, Titusville NJ*

The technology transfer process for analytical methods can be very challenging for Quality Assurance laboratories. Failures in the analytical method transfer process can hold up the implementation of critical testing at a new production site, or delay the start of a stability study needed for approval of a product. In the increasingly competitive environment of pharmaceutical manufacturing, transfer delays cost money. The need for an analytical method transfer to go smoothly and meet the critical path established by launch teams is more important than ever before. Transfer delays or failures are caused by many different variables. A few of the problems encountered in methods that are not rugged, are incomplete sample preparation descriptions, instrumentation limitations and training.

In an effort to create analytical methods that satisfy both submission requirements and end user needs in a Quality Control environment, some laboratories have tried co-development. However, co-development has not been successful at dealing with all of the roadblocks for smooth technology transfer. In this presentation a well-defined process will be described that brings laboratories together in technology transfer. A procedure that enables the end user an opportunity to provide input into method development prior to validation will be discussed.

### Transfer of automated methods within a pharmaceutical research & development environment

*Ronnie McDowell, Ronald Muenz, John Troisi, Stephen Scypinski, Analytical Chemistry Research & Development, The R. W. Johnson—Pharmaceutical Research Institute, Raritan, NJ*

The transfer of automated methods to pharmaceutical quality control laboratories has become a fairly routine process. With the advent of the 'workstation' types of automated instrumentation, the transfer may only require electronically installing the new method from a 3.5″ floppy disc. However, transfer of an automated method into a research laboratory or a contract laboratory proves to be a bit more involved. In these types of transfers the automation equipment itself, which was used to develop the method, may have to be transferred to the receiving laboratory. If not, new equipment may have to be purchased, installed and qualified. Afterwards SOPs are written and the analysts are trained in the use of the new equipment. Once the transfer process has begun any changes in the method requested by the receiving lab have to be addressed. This presentation will discuss the steps involved in this type of method transfer to a research stability laboratory.

### A new concept for the on-line storage of sample plates, integrated with plate preparation for high-throughput screening and 'Cherry-Picking'

*Jacques van den Broek, Will Kuijpers and Thijs de Boer, NV Oragon, Oss, The Netherlands; and Jonathan Connick, Organon Laboratories, Newhouse, UK*

Recently Oranon installed automated screening and plate preparation systems for its research facilities in Oss (The Netherlands) and Newhouse (UK). These robotic systems have been developed in close collaboration between Organon and Scitec Laboratory Automation (Lausanne, Switzerland).

Each of the systems consists of three linear track robots, one of which performs the screening process using standard peripherals. The other two robots take case of the plate preparation and 'cherry-picking' procedures. To this end, copies of our total mother plate collection are stored under controlled conditions in Scitec plate stackers (AutoStack) that can be addressed by one of the two robots. The system is designed in such a way that the loading and refreshment of the on-line storage, screening-plate preparation, and 'cherry-picking' can be executed automatically in 24 hours operation. A more detailed design of the system and the rationale behind it will be further disclosed.

### High throughput extraction and fractionation of samples using Zymate robot and dionex accelerated solvent extractor

*Ramasamy Tamilarasan, Paul Morabito, and Jon Zieman, The Dow Chemical Company, Midland, MI, Ted Weglarz, Dow AgroSciences, Indianapolis, IN, Paul Hazelwood, The Dow Chemical Company, Sarnia, Ontario, Canada, And, William Gee, The Dow Chemical Company, Freeport, TX*

Automated robotics systems were developed and implemented in Dow AgroSciences (DAS), Indianapolis, to generate 400 sample extracts per day and separate each sample extract into 3 fractions. The extraction systems are based on Zymate XP Robot and the samples are extracted in a Dionex accelerated solvent extractor (ASE). Each extraction system includes two ASE and generates 200 sample extracts per day. Two different types of fractionation systems were developed based on the need and availability of automation peripherals. Finally, a fractionation system was developed based on Zymate XP and BenchMate robots to fractionate 100 sample extracts per day by performing liquid-liquid extractions. The extracts are then transferred into 2mL glass vials for analysis. This system was used for developing and optimizing the extraction, fractionation and analysis procedures. In order to maximize the throughput and minimize the number of systems needed, another fractionation system was developed based on Hamilton MPH-96 workstation to fractionate 96 samples at a time in a titre plate by performing liquid-liquid extractions. The sample extracts are then transferred into three 96-well titre plates for analysis. The original sample extracts are transferred into the 96-well titre plate for fractionation by using a TECAN MiniPrep-50 in the extraction system. Barcode labels are used on extraction cartridges, glass vials, and titre plates to track the sample information. Sample preparation data are automatically transferred to a dedicated LIMS system.

### Reformatting equipment for various HTS platforms

*Thomas James Hatcher, Pharmacopeia, Inc., Princeton, NJ*

Recent advances in high-throughput screening have necessitated the development of instrumentation capable of handling multiple microtitre plate formats on the same platform. This includes 96, 384 and 1536-well formats from different manufacturers. Pharmacopeia's internal research combined with multiple collaborative and customer oriented screening efforts against the 6,000,000+

compound library, generated the need for efficient reformatting between different plates. In response to this challenge, Pharmacopeia's HTS and Research Engineering groups have developed a complete family of reformatting tools. This presentation will describe some of the internally implemented equipment and techniques. Among the presented instrumentation will be the industry's fastest universal Single Channel Reformatter (patent pending), the Manual 96—1536 Reformatter, and a fully automated reformatting system capable of handling all types of plates, as well as related liquid dispensing, washing, filling and other tasks.

**Automation and organization for the combinatorial chemistry/medicinal chemistry interface**

*Bob Kennedy, Parke-Davis Pharmaceutical Research, Ann Arbor, MI*

As libraries of compounds have become commoditized, Combinatorial Chemistry groups in large pharma have shifted their focus from library generation to lead optimization. Here, the marginal returns on application of combinatorial techniques to medicinal chemistry projects can be quite high. However, success is often determined by the ability to shift focus from synthetic route development, production techniques, diversity and large numbers of compounds to an emphasis on accessibility of automation, purification and turnaround time of project arrays. This talk will focus on both the technology and organizational structures that leverage medicinal chemists.

**Approaches towards integrated high throughput synthesis, analysis and purification**

*Shahzad Rahman, Discovery Chemistry Europe, SmithKline Beecham Pharmaceuticals, Harlow, Essex, UK*

Traditionally chemists working in the pharmaceutical industry have synthesized a single compound at a time for biological testing. This approach is by its very nature extremely slow and costly. The need to accelerate the drug discovery process has led to the creation of high throughput methods that allow the synthesis of large numbers of compounds either as mixtures or by parallel synthesis of single compounds. Routinely, robotic chemical synthesizers are used for manipulation and synthesis of multiple compounds. Using these techniques hundreds to thousands of compounds can be synthesized and subsequently analyyed and purified in the time taken to prepare just a few analogues by orthodox methodology.

The presentation will describe strategies at SmithKline Beecham for high throughput compound production and processing. In one approach, compounds are synthesized in a semi-automated mode using the IRORI directed sorting methodology. In a second approach, each component of the high throughput process has been automated using specialized robots. Large-scale synthesis, of proprietary diversity reagents, is achieved using the Zymark solution phase synthesizer. Synthesizers (e.g. Myriad, Bohdan RAM, etc.), capable of carrying out complex and demanding chemistry, are used for high throughput library production. Equipment for rapid down stream processing (e.g. l/l extraction), sampling, purification and analysis have also been automated. The systems used at each stage of the process will be discussed using examples to demontrate their capabilities. The presentation will also highlight key issues and challenges in combinatorial technology such as hardware control, data handling and other bottlenecks.

**Fast synthesis at the Janssen Research Foundation**

*Davy Petit, Marc Schroven, Janssen Research Foundation, Belgium*

This presentation gives an overview of how parallel synthesis is performed in the Fast Synthesis Lab, a subunit of the combichem group at the department of medicinal chemistry. The entire workflow and dataflow are automated. A Zymark robot is used for lab-intensive manipulations. An automated preparative LC/MS purification system delivers compounds with a minimum purity of 90% at an average yield of 50 mg. A high throughput analysis tool determines the purity of the compounds based on MS and UV data. Data handling (calculations, registration) is accomplished using MS Excel, Accord Combichem and Accord for Excel.

**Increasing throughput of LC–MS–MS bioanalysis: shortening chromatography versus multiplexing**

*Mohammed Jemal, Bioanalytical Research, Metabolism and Pharmacokinetics, Bristol-Myers Squibb Pharmaceutical Research Institute, New Brunswick, NJ*

With the recent advances made in sample preparation for analysis of biological samples by LC-MS-MS, the sample extraction time has been reduced to less than 30 seconds per sample. Consequently, a chromatographic run time of even two min now appears to be comparatively long. Therefore, there is a renewed impetus to either shorten the chromatographic time or to otherwise achieve analysis of multiple samples within the same chromatographic time through some sort of multiplexing. This presentation will deal with the pros and cons of the different approaches to increasing sample throughput by LC-MS-MS bioanalysis.

**An automated system for rapid determination of semi-optimized MS/MS conditions for bioanalytical mass spectrometry**

*Kevin Whalen, Katrina Rogers, Mark Cole and John Janiszewski, Candidate Enhancement Pfizer Central Research, Groton, CT*

The process of high-throughput ADME screening can generate thousands of samples for analysis in a very short timeframe. Before quantitative LC/MS analysis can be applied to the new chemical entities (NCE) entering these screens, valid MS ion-monitoring conditions must be determined for each compound. We have developed an automated system for the rapid unattended determination of semi-optimized MS and MS/MS conditions (e.g. precursor ion, product ion, polarity, and collision energy) for new chemical entities. The system features a custom software application that coordinates user defined parameters, such as, plate number, well ID and collision energies to be scanned, along with control of the associated sample introduction hardware. The system uses two flow injections of each analyte to determine the semi-optimized MS and MS/MS conditions. The first injection is used to determine optimal polarity and a precursor ion using a rapid Q1 scanning protocol. The Q1 scan is reviewed automatically and the logical precursor ion is chosen relative to expected mass ion ($[M-H]^-$ and $[M+H]^+$). The precursor ions are then used to programmatically build injection sequences for product ion scanning. A color-coded graphical interface is used to facilitate data review. Any unexpected precursor ions obtained during Q1 scanning, and/or suspect ion transitions are flagged and corrected at this time. The data review component of the system has selection criteria built in that can recognize common adducts or losses and flag transitions below a pre-defined intensity threshold. Complete MS and MS/MS conditions are obtained for 96 compounds within 60 minutes and the resulting data are available as injection sequences for direct import into the Sciex sample control function for a quantative LC/MS run.

### Integrating automation and LC/MS for drug discovery bioanalysis

*David T. Rossi, Bioanalytical Core Group, Department of Pharmacokinetics, Dynamics and Metabolism, Pfizer Research, Ann Arbor, MI*

A novel, integrated approach for automated sample handling in drug discovery bioanalysis is described. The process includes aspects of animal study design, biological sample collection, sample processing and high throughput API LC/MS operating in under multiple reaction monitoring (MRM). A semi-automated 96-well liquid-liquid extraction technique for biological-fluid sample-preparation was developed and used in conjunction with the integrated sample handling approach. One plate of samples could be prepared within 1.5 hr compared to 4 hours for a manual approach, and the resulting 96-well plate of extracts was directly compatible with the LC/MS. Feasibility studies for development of the process included sample collection map generation and information management, sample collection formatting, evaluation of alternative dilution schemes for high concentration samples, choice of biological fluid, and evaluating the capabilities of the two liquid-handling workstations. Numerous comparisons between the new approach and conventional sample handling approaches gave equivalent drug-quantitation results for several example compounds. This new sampling process has approximately doubled the efficiency (as measured by studies assayed per month) of drug discovery bioanalysis in our laboratory. The approach was also used in conjunction with time-of-flight mass spectrometry instrumentation (LC/TOF/MS) to quantify and characterize the disposition of simultaneously dosed example drug compounds in rats. Likely strategies for future automated sample preparation workstations are described.

### Integration of new technology for increasing throughput in new drug discovery using HPLC-API-MS/MS: strategies and issues

*Walter A. Korfmacher, Department of Drug Metabolism and Pharmacokinetics, Schering-Plough Research Institute, Kenilworth, NJ*

HPLC combined with atmospheric pressure ionization (API) tandem mass spectrometry (MS/MS) has become a very useful tool in the pharmaceutical industry. The technique of HPLC-API/MS/MS has become very important for both drug discovery and drug development programs. Because of combinatorial chemistry as well as the demand for higher throughput of drugs in the discovery stage, there is a need for techniques that can be used to increase sample throughput in the analysis of samples from pharmacokinetic (PK) studies of new compounds; i.e. what is needed is high-throughput pharmacokinetics, HTPkS. One of the bottlenecks to HTPkS is the PK sample assay step. Strategies for increasing throughput will be described. Bottlenecks that are preventing higher throughput will also be discussed.

### Qualification of a Zymark MultiDose automated dissolution workstation for dissolution testing of fexofenadine HCL capsules

*Tom A. Steinman, Aventis Pharmaceuticals, Inc., Kansas City, MO*

A manual, multi-point dissolution test for powder filled capsules was transferred to an automated dissolution method utilizing the above systems. Both methods require an HPLC final analysis.

Several MultiDose system parameters were evaluated to ensure product result integrity and equivalency. These included carryover studies, line flush studies, vessel wash studies, filter studies, and sample evaporation studies. The most critical evaluation was the statistical analyses (equivalence) of the product results from both methods.

### Automation in pharmaceutical analysis-fiber optic dissolution

*Abe Kassis, Kevin C. Bynum, Lane Gehrlein, Philip Palermo, Purdue Pharma L. P., Ardsley, NY*

A novel UV *in-situ* detection testing methodology has been developed for the analysis of dissolution profiles, for solid pharmaceutical dosage forms. The system utilizes 12 dip type UV probes to monitor the amount of active component releases during a dissolution test. Each probe is placed inside a dissolution vessel and remains in the vessel for the duration of the test. Each probe is then coupled to a spectrometer by means of a fiber-optic light guide. A total of twelve PDA spectrometers are utilized to collect an absorbance spectra from each probe, at set time intervals, during the dissolution test. After setup, the system runs without analyst intervention for up to 72 hours. All calculations and results are automatically calculated, and secured in real time. This publication presents data from a number of pharmaceutical dosage forms, generated in a production environment. The validation and calibration of the system and software will also be presented.

### Expediting the formulation development process with the aid of automated dissolution in analytical research and development

*Jon P. Sadowitz, Barr Laboratories, Pomona, NY*

The development of drugs in the generic pharmaceutical industry is a highly competitive arena of companies vying for few products that are coming off patent. Companies that have been successful in this arena are those that have met or surpassed the critical timeline associated with trial formulation development, analytical method development and submission batch manufacturing and testing.

Barr Laboratories has been successful in the generic pharmaceutical industry for several reasons, one of which includes automation. The analytical research and development department at Barr employs the use of automated dissolution early in the lifecycle of a potential product. This approach dramatically reduces the 'Time To Market' on average for a number of products. The key to this approach is the network infrastructure of the formulation and analytical research and development departments. At Barr Laboratories Inc., our cooperative ability to work and communicate together has driven the departments to streamline and matrix their work efforts and optimize resources and time.

The discussion will overview how Barr Laboratories Inc. has been successful with automation and give a case study of products that have moved with rapid pace through the development cycle.

### Evaluation of an automated Vankel type VII apparatus for testing of extended release OROS® tablets

*Kalpana Chaturvedi and Karen Chang, ALZA Corporation, Mountain View, CA*

An automated Vankel USP type VII dissolution apparatus has been evaluated for testing of extended release OROS® tablets. Reproducibility as well as sample solution evaporation rate, homogeneity, and carryover were studied using ALZA OROS® tablets. Performance of this automated tester was also compared to the ALZA USP VII bath that requires several manual sample preparation steps. The OROS® tablets were released for 10 hours at 37 °C, and the released sample solutions were collected via a Vankel 8000 collector. Sample solutions were then analyzed using a validated isocratic HPLC method. Homogeneity of the sample solution was evaluated by manually removing the sample solution from the top, middle, and bottom of the same sample tube. Reproducibility was evaluated by measuring the release rate of six tablets per day on 3 separate days. The data showed that both ALZA and Vankel VII apparatus exhibited similar release-rate profiles for the OROS® tablets studied. An evaporation study showed very minimal evaporation during the 10-hour release interval. The sample solution was found to be homogenous, thus allowing automated transfer from the sample tube to the VK 8000 collector. Carryover from one interval to the next was insignificant (1%) and similar for both ALZA and Vankel release-rate testers. Release-rate profiles from all three reproducibility sets were very comparable. It is concluded that the Vankel VII bath with automated collector can be employed for release-rate testing of ALZA OROS® tablets.

### BioPrint™: the leading edge in drug profiling—optimizing the hit to lead strategy

*Michael Entzeroth, CEREP, Rueil-Malmaison Cedex, France*

Modern drug discovery produces an increasing number of lead and development compounds with a vast number of related results, triggered by the installation of modern technologies, such as combinatorial chemistry and (ultra) high throughput screening ((u)HTS). Lead selection, to initiate successful discovery and development programs, has become more difficult and sometimes a matter of serendipity. As a consequence, the pharmaceutical industry has put emphasis on the development and automation of secondary tests. High throughput profiling using robotics and automated workstations allows us to accumulate a broader knowledge on the hits identified in the primary screening. These data give rise to a more in-depth analysis of the compound profiles with respect to their pharmacological and pharmaceutical properties. The avaluation of these profiles provides knowledge-based decision criteria by distinguishing selective from promiscuous candidates and clustering candidates and even targets in hierarchical families. BioPrint™ is a data mining approach which generates those models that correlate molecular features of a compound to patterns of *in vitro* biological activity and patterns of *in vitro* activity with *in vivo* biological activity. This data mining opens a new approach for candidate selection and early discovery activities, such as target identification or library design and will result in the long term cost savings due to reduced failure rates and time spent on the research and development process.

### Enhancing drug discovery: utilization of V*I*TA™ fluorescently labelled ligands in high throughput capillary electrophoresis screening

*Yuriy Dunayevskiy[1], Tom Riera[2], Paul Gallant[2], Dallas Hughes[1] and John Finn[2], [1]Cetek Corporation, [2]Cubist Pharmaceuticals, Inc*

The rise in bacterial resistance has lead to an increased emphasis on target-based methods for antibacterial discovery. An HTS assay is a key component in most target-based strategies. For many targets, however, development of a HTS assay is difficult and requires considerable time prior to initiation of screening. The V*I*TA (Validation *In vivo* of Targets and Assays) process validates bacterial targets and generates a peptide ligand that is used in fluorescent polarization (FP) HTS assays. This talk will illustrate the use of a novel capillary electrophoresis (CE) screening technology applied to a V*I*TA validated target and peptide. A fluorescent CE-HTS binding assay was developed and optimized in less than 1 week. The CE assay required significantly less material than the FP assay and increased throughput by allowing the screening of mixtures. Details about the CE-HTS assay performance will be presented.

### Automated high throughput capillary electrophoresis (HT-CE) screening

*Dallas E. Hughes, Herbert J. Hedberg, James N. Little and James L. Waters, Cetek Corporation, Marlborough, MA*

A new screening technology, based on capillary electrophoresis, is being widely accepted as an alternative to conventional screening assays. HT-CE identifies target-binding ligands in synthetic compound and natural extract libraries. The assay can rapidly rank 'hits' by binding affinities and can distinguish between different target binding sites. Assay development is easy and fast with no requirements for competitive ligands, radioactive reagents, antibodies, or knowledge of the target's biological function.

Since no high throughput CE instrumentation was available, this talk will describe new automated instrumentation and capabilities for performing HTS by CE. Examples of various assays and assay formats will be described using this new instrumentation.

### The Luminex LabMAP™ system: a rapid, homogeneous multiplexed assay platform

*Lekha Patel, Kerry Oliver*, Janet Davis, Brian Magliaro, Lee Peligrino, Scott Wadsworth, John Siekierka, Alex Collins, Robert Zivin. The R.W. Johnson Pharmaceutical Research Institute, Raritan, NJ, *Luminex Corporation, Austin,TX*

The Luminex device combines fast, microsphere-based assays with the ability to carry out parallel reactions in single wells The LabMAP system is a multiplexing technology capable of rapidly analyzing up to 100 different analytes/reaction products in a single sample. Assays are developed in a homogeneous format on unique sets of 'fluorescently bar-coded' microspheres and quantified by the fluorescence intensity of a reporter ligand. Most assays formatted for microtiter-based assays are amenable to miniaturization and rapid transfer to the bench-top LabMAP system. These include: immunoassays, enzymatic assays, transcriptional profiling, and receptor binding assays. Of particular note are the Insulin-induced receptor phosphorylation (a whole cell system) and the 'multiplexed' cytokine assays. Assay details and results will be presented.

### Xpress-Screen™ mRNA detection direct quantitation of specific mRNA in cell lysates in an automated, high-throughput format

*Kimberly Spofford, Applied Biosystems—Tropix Division, Bedford, MA*

Monitoring changes in gene expression levels is the most informative and rapid approach to date for evaluating the effects of potential drugs on the cell. An ideal HTS format of that approach should provide reliable information output in a cost-effective manner. Current methods such as a reporter gene approach, quantitative RT-PCR, and other mNRA detection assays do not adequately fulfill these criteria. A novel assay for precise and direct quantitation of specific mRNA in cell lysates is presented. This method is based on the detection of multiple-biotinylated long single-stranded DNA probe/mRNA hybrids captured on a streptavidin-coated microplate. Detection of the hybrid by a specific alkaline phosphatase-conjugated antibody features several levels of signal amplification. The assay is adapted for use on the Allegro™ UHTS system (Zymark Corporation, Hopkinton, MA). This method allows for rapid assay development in any cell type including yeast, multiple-gene readout in 384-well plates, and detection of 1-2 copies of mRNA per cell. In contrast to reporter gene systems the method reflects not only native regulation of synthesis but also the rate of degradation of the mRNA in response to the signal. Different applications in monitoring the cellular regulatory network using this method are developed.

### Screening in the 21$^{st}$ century: quality data with UHTS speed

*Carol Ann Homon, Mohammed A. Kashem, and Richard M. Nelson, Boehringer Ingelheim Pharmaceuticals, Inc., Ridgefield, CT*

In the early days of high throughput screening, the quality of data produced was often compromised by inherent limitations in available automated devices and assay biochemistry. What worked well in manual assays on the bench did not perform at the same level in the unforgiving environment of the automated screen. Advances in technology have completely reversed this situation. State-of-the-art liquid handling, robotics, and detection technology, together with new HTS-enabling assay biochemistry, have elevated the data quality of ultra-high throughput screens above that which can be achieved using manual assays and older methods. The major force that has propelled this quiet revolution in screen quality is the advent of assay biochemistries designed specifically for HTS and UHTS. Clear leaders in this arena are FRET, TR-FRET, and FP homogeneous, or mix-and-read, formats, whose spectacular performance in ultra-high throughput screening is unrivalled by radiometric, immunofluorescent, or immunoenzymatic methods. This talk will review our experience with DELFIA, LANCE, FP, and radiometric UHTS run on the Zymark Allegro robotic system, prosecuting several different molecular targets. We will describe certain fluorescent screens that have produced $Z'$ values as high as 0.9 and Z values of 0.7 across run sets of several hundred plates.

### Workstations versus fully automated systems: pick your poison

*Rich Harrison, DuPont Pharmaceuticals, Wilmington, DE*

A topic that has been raised at many an automation meeting and bar room debate is the resolution of the age old question: it is better to equip a high throughput screening lab with several small, dedicated workstations, or to run with a fully integrated automation system?

While small workstations are easy to operate, they lack the ability to perform laborious, complex procedures without manual intervention. Fully automated systems, while able to process large numbers of samples in less time, have been plagued by down time. Simply put, the more things to break the more things will break. While the arguments are passionate for both sides, the choices are most often made by economic, rather than practical and scientific criteria. This talk will focus on these often overlooked practical and scientific criteria. Examples of real world assays performed on both workstations and fully automated systems will be compared in terms of ease of use, reliability, and performance.

### High-throughput assay and analysis strategies for pre-clinical ADME/Tox screening

*Jeff Whitney, Novatia Corporation, Yardley, PA*

In recent years much progress has been made in the drug discovery areas of the pharmaceutical industry towards automating the synthesis and functional screening of small molecules. Hundreds of thousands of compounds can be synthesized and screened using combinatorial chemistry and high-throughput functional screens in a fraction of the time previously required. However, as many discovery scientists are now realizing, quantity alone does not fill the drug development pipeline with compounds more likely to reach the marketplace: an increasing emphasis on quality is emerging. This greater emphasis on quality requires smarter decisions for library design and lead candidate selection by incorporating ADME/Tox tendencies that shed light on a compounds *potential* 'drugability'. Two major approaches are evolving for obtaining pre-clinical ADME/Tox information: in-silico computer algorithms which attempt to make predictions based mainly on molecular structure, and *in-vitro* assays which monitor potentially relevant biochemical transformations and interactions. This talk will focus on assay design and detection strategies for *in-vitro* screens used to physically measure ADME/Tox parameters such as metabolic stability, permeability, solubility, inhibition, etc. The use and benefits of high-throughput LC/MS as a direct structural detector for these assays will be highlighted. Emphasis will also be placed on issues of sample preparation, automation and throughput.

### ADMET profiling of combinatorial libraries and lead optimization for drug discovery

*E. Priya Eddy, Millennium Pharmaceuticals, Cambridge, MA*

Estabishment and validation of high throughput ADME/TOX assays is extremely important for 1) future library design; 2) validation of the medicinal relevance of the libraries and 3) lead optimization and selection of drug discovery candidates.

A battery of ADME/TOX assays consist of: Aqueous solubility, Metabolic stability, CYP-450 Inhibition, Intestinal permeability, Cytotoxicity and cell proliferation assays and Protein Binding measurements. About 4000 library compounds (10% of libraries) passed through each of these assays (except protein binding under validation) that have been automated both for liquid handling and data processing. Details will be discussed.

Aqueaous solubility at 10uM and 100uM was evaluated in a 96-well plate by using a UV spectral scan from 190–300nm. Metabolic stability of compounds at 10uM in human liver microsomes +S9 in the absence and presence of NADP was evaluated using modified Gentest methods. Fluorescence based CYP-450 Inhibition assays at 10uM compound concentrations were evaluated which include 4 major P450's: 1A2, 2C9, 2D6 and 3A4 (modified Gentest methods). Interstinal permeability assays were run using Caco-2 cell cultures (18–25 days) in a 24-well plate format. Compound permeability (100uM) was evaluated in the basolateral-to-apical direction. Cytotoxicity and cell proliferation assays were run using sub-confluent MDCK cell cultures and incubation of library compounds (100uM) for 24 h. Cellular viability and proliferation were determined by using XTT reagent (Roche).

Results: Throughput for each of the assays was between 800–120 compounds/week/person except for permeability assays (300 compounds/week). A database containing such ADME/TOX properties for compound libraries will be crucial in SAR for future library design.

### Plasma pooling and continuous blood withdrawal methods to increase throughput for *In Vivo* pharmacokinetic screening

*Cornelis E.C.A. Hop, Qing Chen, C. Keohane, Zhen Wang and Cloria Kwei, Merck Research Laboratories, Rahway, NJ*

Due to the amount of combinatorial chemistry and other parallel synthetic approaches, the number of compounds that have to be screened pharmacokinetically *in vivo* has increased dramatically. Automation of several steps during the extraction and analysis has been employed to increase throughput. Alternatively, mixtures of several compounds can be dosed to animals followed by analysis of the samples by LC-MS/MS. Using this approach introduces the possibility of drug-drug interactions. To circumvent these disadvantages individual compounds can be dosed, followed by pooling of plasma samples of animals dosed with different compounds. As an alternative we investigated a method in which appropriate aliquots of the plasma samples obtained from all time-points are pooled, yielding one sample that has a concentration proportional to the pharmacokinetic area-under-the-curve (AUC). Using this procedure there is only one sample per animal instead of 8–10 samples. Because the animal receives only one compound, pharmacodynamic information can still be obtained and drug-drug interaction is not a concern. Of course this approach can also be combined with cassette dosing. In the latter case, one sample will provide the AUC for all compounds. This pooling method represents a fast screening tool in the discovery phase of drug development and is probably most useful when plasma AUC values after oral and/or intravenous dosing are the main concern of the discovery program. Nevertheless, this pooling method still necessitates a considerable amount of pipetting. Therefore, we investigated continuous blood withdrawal to obtain one sample for each animal.

### A clinical trial on a plate: the potential of 384 format SPE for high throughput bioanalysis using LC-MS-MS

*Robert Biddlecombe and Steve Pleasance, Glaxo Wellcome, Park Road, Ware, UK*

The benefits of moving to higher density plates (e.g. 384) have already been established in HTS applications, e.g. less reagents, faster screening, smaller sample volume. In this work we evaluate the potential of higher format SPE for bioanalysis using LC-MS/MS.

Project compounds with existing, validated SPE-LC-MS/MS methods in the 96 well format were selected for the work in order to identify the advantages in moving to a higher, denser format. As there are no commercially available 384 well format SPE blocks, several prototype microtitre plates were manufactured for this work.

All liquid handling was automated using a Packard MultiPROBE II robotic sample processor and a 96/384 Rapid plate. Solvents for conditioning, washing and eluting were drawn through the SPE block using centrifugation. The SPE extracts were analysed by LC-MS/MS using HP1100 pump, a CTC LC PAL auto-sampler and a PE-Sciex triple quadrupole mass spectrometer using a TurboIonspray interface and selected reaction monitoring.

The Pros and Cons of 384 SPE will be discussed. These include:

- reduced sample volume;
- validation;
- the potential for cross well contamination;
- the ratio of calibration standards to unknown samples;
- time required for sample preparation and overall analysis time (384 versus $4 \times 96$);
- costs of consumables and re-tooling for peripherals;
- extract stability.

### Managing the challenge of efficient sample distribution in an international company with different research sites

*Gerhard Mihm, Lead Discovery, Screening Support Team, Boehringer Ingelheim Pharma KG, Biberach, Germany*

In the last few years the increasing throughput in HTS and CC has put an enormous pressure on the sample management labs (dispensaries) to make all samples available to the HTS groups, the biological homelabs and analytical groups in an enormous format and on time. In addition, efficient exchange of the necessary sample data is essential for rapid testing and reporting of the results. This situation is particularly difficult in companies with many research sites and hence different destination of shipments.

BI is a worldwide operating pharmaceutical company with research sites in Europe and America. Compound collections are maintained in Germany and the US, while biology is done at all research sites. Samples are distributed from the compound stores to the sites on a regular basis.

We have developed a strategy enabling an efficient worldwide sample exchange based on the shipment mainly of solutions. In the BI dispensary in Biberach several workstations for the solubilisation of the samples and aliquotting the solutions into plates and tubes for individual sample access are available. In addition, the vast number of tubes are handled with an automated sample storage and retrieval system that has been implemented together with REMP.

The exchange of the associated data is done via direct database links and a worldwide data turntable based on ORACLE technology. Details about our strategy and the automation established will be presented.

### A survey of the various plate sealing technologies

*Michael Routburg and Carol Ann Homon, Boehringer Pharmaceuticals, Inc., Ridgefield, CT*

High throughput screening is dependent upon the quality of its samples stored as DMSO solutions. The storage and shipment of liquid libraries in plate format requires a proper sealing method which will ‘contain’ the solution within its well (isolated from the neighboring compounds) upon thawing. The seal must not introduce any impurities into the solution either by direct contact of the DMSO with the seal or by indirect contact (volatile components).

The results of an evaluation survey of the various sealing methods (cap mat, adhesive, heat and gasket) and the present commercial devices will be presented and compared. Advantages and disadvantages of each will be highlighted. Automation options associated with the various methods will also be discussed.

### Optimizing production of serially diluted compounds and distribution to multiple targets

*Cole O. Harris and Stephanie L. Schweiker, GlaxoWellcome, Inc., Research Triangle Park, NC*

The need for a multiple-target compound selectivity program led to the establishment of a single robotic system that produces a compound’s serial dilution and its distribution to multiple replicate assay plates. A Tecan integrated into a Zymark robotic system produced the serial dilutions and the subsequent replicate assay plates were produced quickly and accurately by an efficient use of the carousels and rapid plate. Currently this process allows for the production of over 200 serial dilution assay plates in a workday.

### Implementation of an automated data transport architecture for discovery analytical instruments over a secure corporate intranet

*Martin M. Echols, Bristol-Myers Squibb, Lawrenceville, NJ*

The dramatic increase in industry utilization of combinatorial chemistry technologies and the explosion in automation for high throughput screening have had a tremendous enabling effect on the pharmaceutical drug discovery process. But these advantages have also brought with them new challenges and foremost of these is the current avalanche of chemical information. This paper addresses a crucial part of this new challenge, the fast, reliable and automated transportation of analytical data from its source in laboratory to its destination, the LIMS or other point of access. An architecture is presented that utilizes intranet network elements commonly available today within most business both small and large. An implementation of this architecture in Microsoft Windows NT using the popular development tool Visual Basic and commercial software components is described with an evaluation of its performance.

**Computational chemistry, data mining, high-throughput synthesis and screening: informatics and integration in drug discovery**

*Charles J. Manly, Neurogen Corporation, Branford, CT*

Drug Discovery today includes considerable focus of lab automation and other resources on both Combinatorial Chemistry and High-Throughput Screening. Computational Chemistry has been a part of pharmaceutical research for many years. The real benefit of these technologies is beyond the exploitation of each individually. Only recently have significant efforts focused on effectively integrating these and other discovery disciplines to realize their larger potential. This presentation will describe one example of these integration efforts. Examples from experiences at Neurogen from the last 6 years will be described.

**Improved effectiveness in mixture screening through better data management**

*Linda Traphagen, Abbott Laboratories, Abbott Park, IL*

Screening using compound mixtures rather than individual compounds can save significant time, and considerably reduce costs. However, identification of active hits in mixtures can be difficult, and can yield ambiguous information. Abbott's internally developed screening database allows complete tracking of all the compounds in these screening wells, along with their associated data, thereby facilitating precise identification of all active compounds while maximizing the cost savings associated with screening in mixtures.

At Abbott we screen mixtures of ten compounds, which originate from an orthogonal mixing of 100 source plates to yield 20 mixed screening plates in 96 well format. Each compound is represented twice in unique mixtures. Hits are designated as one of two types: 1) matched 'hits' and 2) unmatched 'hits'. Matched hits are those where the compound has shown activity in both its respective wells. Unmatched hits show activity in only a single well. In the latter case, there is no corresponding active well to indicate which of the ten possible compounds in the active well is responsible for the well's activity.

Confirmation of activity is done as single compound tests. Retests are performed on all reported matched compounds and on the ten compounds from any unmatched wells. When a matched compound fails to confirm in retests, the two wells of the original matched hit are demoted to unmatched status. This process thus identifies an additional eighteen compounds making them available for retests. This re-iterative testing process enables us to identify all active compounds in a mixed compound screening format.

**Chemometrics in high throughput systems—utilization issues and strategies in using multivariate data analysis in integrated robotioc systems and Lab-on-Chip analyses**

*Richard P. Gill, Veritec, Inc., Essex, CT*

High Throughput Screening (HTS) incorporating multiple tests and instrumentation within the same system generates reams of multivariate data that can be difficult not only to process but more importantly to interpret. Utilization of chemometrics in the analysis of multivariate HTS data sets can provide substantial analysis benefits.

Chemometrics is a broad field that can be defined as the application of mathematical and statistical techniques to chemical data. This discipline uses mathematical and statistical methods to: (1) extract out the maximum amount of information from large multivariate 'chemical' data sets, and (2) design or select optimal measurement procedures and experimental methods.

The presentation provides a nonmathematical overview of the challenges in building and operating HTS systems incorporating multivariate data analysis. Actual data examples, issues, and strategies stemming from the use of this approach in HTS and Lab-on-chip systems will be discussed.

**Managing laboratory automation in a contract laboratory**

*Darren Jantzi, Stability Manager, PPD Development, Middleton, WI*

Automation provides contract research organizations (CROs) a medium to provide high volume, compliant analysis to clients. Evaluating and selecting automation that is worthwhile transferring to a contract site is an important process in a successful program.

Stability testing for finished pharmaceutical products lends itself to automation in a contract environment. There is a large volume of repetitive work extending up to 5 years. If automation is managed properly it can accelerate bringing a drug to market through more consistent and timely analysis of stability data.

Automation also brings with it a host of challenges: cost, technology transfer, maintenance and calibration, IQ/OQ/PQ, data organization, and more. However, the benefits derived from automation, including increased efficiency, quality, and productivity outweigh these potential challenges.

This talk will focus on a contract lab's experience with implementing and managing automation through case studies.

**Automation for contract laboratories—benefits from a customer perspective**

*Nigel North, SmithKline Beecham Pharmaceuticals, Harlow, Essex, UK*

Automation requires significant capital investment and for contract laboratories this funding may be difficult to justify. However, with increasing commitment to the automation of dosage form analysis by pharmaceutical companies and the adoption of automation earlier in the development process, outsourcing of automated methods will be an important requirement in the future. Contract laboratories that do not have automation equipment will not be able to compete effectively for business as those that have this capability. The benefits to the customer of outsourcing automated methods will be discussed together with the potential problems for the contract laboratory in forming a strategy to meet this need. The adoption of new technology in other development functions (eg. bioanalysis) by pharmaceutical companies and contract facilities has been very successful. The learnings from this situation will also be briefly outlined to provide some perspective around the implementation of automated methods.

**An automated approach to the supply of samples for screening**

*Terry Wood, Medicinal Technologies, Pfizer Ltd., UK*

Some years ago the Drug Discovery teams at Pfizer Ltd. In the U.K., predicted that sample supply would become a rate-limiting factor in successful implementation of high throughput screening programs. A team of scientists was assembled to develop the concept and specification of an automated system that would securely store our rapidly growing sample bank, and supply test plates to an intensive program of primary and follow-up HTS.

Following competitive tender, a Manchester based company, RTS Thurnall Ltd., was selected to design and build an Automated Liquid Sample Bank (ALSB) at Pfizer's U.K. base in Sandwich, Kent. An intensive program of joint meetings, acceptance testing and integration with Pfizer's existing systems culminated in the launch of the ALSB on April 12$^{th}$ 2000, fully automating the process of liquid sample ordering and delivery.

The system contains DMSO solubilized samples stored at $-20\,^{\circ}C$ in microtitre formatted racks of tubes, and is capable of assembling collections of up to 6000 individually selected tubes or 500 racks of tubes in a 24 hour period. This period includes time to thaw the samples, aspirate them and dilute them to the concentration requested. The scientist placing the order does so from his/her desktop PC, specifying the compounds to be screened and details of concentration and dilutent required. From commitment of this order, the process is entirely automated until the ALSB operators unload the completed test plates for delivery.

The key hardware components of the system are a freezer store with a capacity of 2.5 million samples, a defrost station, and twin liquid handling cells all connected by a transport system of infeed and outfeed conveyors. The whole system is controlled by an order processor, which directs all decisions on the most efficient use of hardware, appropriate dilution algorithms and also controls the various administration functions available. Orders are created using updated versions of sample ordering and experiment creation software already in place at Sandwich, with which our scientists are familiar. A vital requirement, prior to final acceptance, was the successful integration of these software packages with the ALSB database. We are now able to support primary HTS, follow up active compounds, and deliver samples to $IC_{50}$ programs at a rate in excess of our current requirements. In addition, we can assemble bespoke selections of compounds in numbers that would have been previously impractical. Adjacent support hardware allows purity checking of stored samples, and reformatting of ALSB output, if required, by the screening robotics.

Following a successful partnership with the system suppliers, RTS Thurnall, the Pfizer ALSB facility is up and delivering. The system forms a vital central component of lead discovery and optimisation operations at Sandwich.

**Automated liquid handling in compound dissolution and distribution robots**

*Kevin Olsen, Wyeth Ayerst Research, Pearl River, NY*

The preparation of highly concentrated DMSO/Compound liquors is the starting point for most drug discovery screening. Because of the physio-chemical properties of the liquors, the frequent presence of undissolved materials, and their importance to the discovery organization, specialized robotics are often required for this task. This presentation follows the development and optimization of automated pipetting devices used in the Research Compound Bank by the Robotics & Automation Department.

**SmartPlate cassette simplifies compound handling**

*Jeffrey A. Karg, Boston Innovation, Inc., Cambridge, MA*

The twin revolutions of geonomics and combinatorial chemistry have created an explosion in the number of compounds for screening. Managing these large compound libraries efficiently is a big challenge. A consortium of industry leaders is guiding development of the SmartPlate Cassette, which integrates shipping, storage and sub-microliter non-contact dispensing of compounds. This approach could be the basis of a new industry standard for compound management. The presenter will highlight implementation examples and current performance data.

**The many faces of the Hamilton Microlab 2200 liquid handler station at Bristol-Myers Squibb**

*Joseph Yacobucci, Benjamin Carvalho, Edward Pineda, Jonathan Severino, James Mongillo, Steven Innaimo, Richard Belval, Bristol-Myers Squibb, Wallingford, CT*

This presentation will describe the many implementations of the Hamilton Microlab 2200 in Lead Discovery over the last 13 years at BMS. Subjects covered will include single, 8, 16, and 48 channel devices, software and hardware, operations into 96, 384, and 1536 well microplates, and use of liquid sensing to replenish reagents on the deck as needed. As a high-speed liquid handler, it was inexpensive, easy to operate, and very flexible, thus making it very useful for original thought. Hardware and software can easily be customized for highly successful use, as a workstation, or in a high-speed fully automated robotic system. Also to be presented are the possible future predictions of new faces for the Hamilton Microlab 2200.

### A unique robotics solution to achieve routine 1536-well cell-based screening

*Daniel Sipes, Novartis Institute for Functional Genomics, San Diego, CA*

Assay miniaturization can reduce screening cost/well while increasing throughput. Obstacles to implementation of miniaturized screening have included high instrument cost and unacceptable reliability or performance of either the instruments or the assays in the 1536 well format. To accomplish miniaturization of cell-based assays, GNF has taken an industrial approach to building a *de novo* screening system. Modification to off-the-shelf instruments resulted in reliable nanoliter dispensing to 1536 well plates. Unique solutions well developed to reduce evaporation and edge effects in 1536 well plates. To maintain versatility, custom compound and assay (cell) plate storage solutions were developed.

### A novel nanoliter fluid transfer system

*Chris Shumate, Mitch Dotktycz, Oak Ridge National Laboratory, Oak Ridge, TN*

A novel, low volume transfer system has been designed and tested in a variety of configurations. The system consists of a precision microfluidics coupled to high-speed valving and pressure delivery. Volumes of 5 nanoliters to multiple microliters can be rapidly transferred in a highly parallel manner. The system can be configured for reagent delivery and sample transfer and offers the benefit of size, economy, and robust performance. Potential uses include library replication, sequencer and synthesizer applications, micro-array production, and as enabling middle-ware for microplates and lab-on-a-chip integration. Various configurations, their use scenarios, as well as performance specifications will be presented.

### 384-well small volume microplates: a new platform for approaching the 2 to 10 μL range?

*Günther Knebel; Greiner Labortechnik GmbH, Frickenhausen, Germany*

The success of the 1536 well plate has encouraged us to investigate other user-friendly plate formats in the 2 to 20 μL range which offer similar savings in reagents at lower investments. A novel 384 well small volume (SV) microplate with a total well volume of 30 μL saves reagents and reduces costs per well significantly without major changes in robotics and liquid handling.

An optimized well shape combined with a properly adjusted Z-axis of the plate reader can improve the signal strength in these small volume microplates. Sensitivity of 384 and 1536 well plates is very similar in the 2 to 20 μL range and enables us to perform homogeneous, heterogeneous, and cell based assays. Various cell-based assays have been investigated in black tissue culture treated plates by monitoring fluorescence in a CyQuant assay.

The data presented compare the properties of 384 SV and 1536 well plates in the sub 10 μL range and highlight the impact of the appropriate imaging systems and plate readers.

### An automated liquid–liquid extraction system utilizing interface detection and supervised by an industrial programmable logic controller

*Stephane Bostyn*, J. Gaucher*, G. Christophe*, R. Soukehal* and C. Porte**: *Laboratoire de Productique Chimique—IUT-Université d'Orléans, Orléans, France, **Laboratoire de Chimie Industrielle du Conservatoire National des Arts et Métiers, Paris, France*

In the case of the development of new products in organic synthesis, the final step of the process must offer the best purity. In this way, the final step is a unit operation of isolation like distrillation, crystallisation or liquid–liquid extraction. Therefore, it is necessary to integrate module of purification on an automated platform for achieving the whole process.

In our case, an automated platform was based on a robotic Zymark architecture allowing us to make sample preparation and the reaction steps. For the purification module, we opted to develop our own home liquid–liquid extraction module. This unit operation permits us to separate the final product in the case of liquid phase peptide synthesis in, but equally, in the case of isolation of vegetal molecules.

The module capacities had to permit the multistage extraction and the determination of the binodal curve for optimising the process, and to possess in own system for liberating the Zymark robot for other tasks. This system is an industrial PLC (Omron) which manages the whole steps of extraction. The PLC is connected by Zymark system by logical inputs and outputs.

For carrying out these requirements and in order to have a low cost, a classic laboratory funnel is used on which an electrovalve is implemented at this bottom. The Zymark arm brings the reactor to the module and quantity of extraction solvent is introduced by MLS. Afterwards, the mixing is made by bubbling. Finally, a capacitive detector allows the detection interface. After this operation, the phases are oriented owing to several electrovalves first for analytical sample and after for storage. In position of storage, a new step can restart in function of the operator parameters.

### Other uses of laboratory automation: statistically designed experiments in organic chemical process research and development

*Gary S. Proehl, Eisai Research Institute, Andover, MA*

With the advent of High-Throughput Screening (HTS), the process research chemist in the pharmaceutical industry has seen two major changes. First, the increased number of lead compounds being generated by HTS is a challenge for the process development chemist doing synthetic route optimization. Second, the multi-position reaction equipment used by HTS is an opportunity for performing parallel chemical reactions. Combined, these changes beckon a new approach to process chemical R&D.

This presentation will describe work using statistically designed experiments performed on a 12-position array reactor. This methodology can rapidly and efficiently find optimal parameter settings for a chemical reaction. Using this approach, chemists in process R&D can address the challenge of an increasing workload while delivering improved chemical reaction conditions.

### Automation in process development: the Pfizer robotic experimental design system (RED)

*Neal Sach*, P. Higginson*, P. Hailey*, A. Pettman*, J. Wallis**, *Pharmaceutical Sciences, Pfizer Global Research & Development, Pfizer Ltd., Sandwich, Kent, **Logic Rapid Ltd., Chestnuts House, West Byfleet, Surrey, UK*

Statistical design of experiments is a simple but powerful tool for planning experimental investigations to extract the maximum amount of information with the most efficient use of resources. The technique is particularly applicable for commercial route optimization, process parameter setting and process robustness where there are huge business benefits to be gained. Until recently however, this approach has been tedious and potentially inaccurate due to the large number of experiments that were performed by hand and the high level of quality required.

Pfizer (UK) Process Research and Development has sought to combine technological innovation with experimental design to facilitate efficient process optimization of late stage candidates. A robotic system was designed, in collaboration with Zymark (UK), to closely model commercial plant capability whilst allowing high throughput of reactions with the additional quality automation offers.

Two plant reactor mimics and peripheral modules were designed and integrated with a Zymark XP Track Robot. Throughput limitations of two reactors were resolved by incorporating automated self draining/self cleaning modules into the system to allow 24/7 operation. LECIS based control software was written in-house allowing simple high quality, reproducible control.

The presentation will focus on the design and development of the system with real examples of the system success as a total automated solution to experimental design.

### Using automation to achieve compliance in R&D and QC laboratories

*Ronald F. Tetzlaff, KMI/PAREXEL, LLC., Alpharetta, GA*

Most R&D and QC laboratories use Laboratory Information Management Systems (LIMS) to capture, analyze, and maintain the large amounts of analytical data that are needed to comply with current Good Manufacturing Practices (cGMP) Regulations. Even with the use of LIMS systems for managing the data, quality professionals face some formidable challenges when trying to establish and maintain systems and processes that are in compliance with the requirements of the Food and Drug Administration (FDA). For example, a large number of personnel must maintain an awareness of regulatory requirements (including changes in policies and expectations), and practical ways are needed for determining whether the facilities, systems and processes have achieved a state of compliance.

A consultant's perspective will be presented about ways to use automation to improve the communication of information about performance of the various systems and processes. This discussion will take into account the communication barriers that are created by the diverse organizational structures, including the conflicting responsibilities that are inherent between R&D and QC departments. Presented will be some practical ways to use automation in ways that will allow responsible management to be aware of the regulatory expectations. Automated tools should be designed to ensure that responsible personnel have available sufficient data about the performance of the systems and processes, and the information must be presented in a manner that key personnel will be able determine whether data and information are in accordance with the regulatory requirements. Automated tools should be designed to ensure that personnel know the boundaries of their responsibilities, and to document the basis for their judgments and decisions. The quality systems used in the healthcare industry have reached a level of diversity and complexity that necessitates new ways of using automation in R&D and QC laboratories.

### Re-testing in the regulated laboratory

*Jean Blackston Hill, U.S. FDA, Philadelphia, PA*

### The Trident™ multiple parallel synthesizer by argonaut technologies as a workhorse in an integrated automation environment

*Richard A. Wildonger, Chemical Technology Section, Enabling Science and Technology Department, AstraZeneca Pharmaceuticals, Inc., Waltham, MA*

Critical to the effective implementation of high throughput methods of synthesis is the necessity for a significant supporting level of automation. There are a number of critical issues associated with the successful introduction, and supporting role, of automation of small molecule chemical synthesis. Clearly there are needs for automation to increase drug candidate synthesis throughput. Automation of repetitive and laborious tasks associated with the synthesis process can release skilled chemists to apply their talents to the more challenging investigational aspects of developing new synthetic protocols. This provides continuity in the compound supply pipeline and ensures an optimal use of the automated platform for compound production. The very high fidelity of performing repetitive processes that can be managed through automation also removes some of the limitations and errors associated with more fallible human operators. This can include very difficult tasks associated with tracking data, and general information and inventory management. Taken collectively, these attributes associated with automation can lead to greater efficiencies, throughputs and improved allocation of human resources with concomitant reductions in costs associated with current day and future drug discovery.

In our library development/synthesis paradigm we feel that automation support must be invoked early in the process and that this automation support must continue throughout the project. One key component of our automated Multiple Parallel Synthesis capability is the Trident™ automated synthesizer by Argonaut Technologies. In this talk we will focus on the role that the Trident™ plays in our Chemical Technology group as libraries are brought to fruition.

### Automation in high throughput chemistry

*Bill MacLachlan, SmithKline Beecham, Essex, UK*

Successful high throughput chemistry in a drug discovery environment requires the strategic use of automation. Pressures are ever increasing to prepare rapidly diverse drug like compounds in good purity to satisfy the demands of high throughput screening and genomics. In addition to aiding the preparation of large collections of compounds for lead generation, automation must be applied to lead optimization to attain fully the efficiencies required in the complete drug discovery process.

The successful automation of medicinal chemistry requires the prudent use of a range of platforms to perform synthesis, analysis and purification of target molecules. The devices and processes must cope with the production of compounds of different physicochemical properties in a variety of scale. Most importantly, the automation must be suitable to be used by the skilled medicinal chemist as a routine tool to exploit fully their creativity and craft.

A discussion of key automated devices and their integration will be presented.

### Extending the range of executable automated chemistries available to the Zymate XP synthesis system

*David A. Rudge, AstraZeneca UK, Macclesfield, Cheshire, UK*

Automated Chemistry has been undertaken in the High Capacity Synthesis Laboratory at AstraZeneca's Alderley Park Research Laboratories using the Zymate$^{XP}$ Synthesis Robots since 1992[1,2,3]. During this time over 80 different synthetic procedures have been undertaken involving over 30,000 reactions.

One of the aims of the High Capacity Synthesis Group, alongside the provision of an automated chemical synthesis service, has been to disseminate chemical technologies into the general synthesis laboratories. This has been ahieved using, in part, the Benchmate™ Work-up System, developed in collaboration between our laboratory and Zymark UK.[4,5]

With an enabling technology available to the general synthetic chemistry community, we have determined to develop the Zymate$^{XP}$ Synthesis Systems to undertake higher capacity synthesis, both in numerical terms and also that of scale.

We have also started to develop the capabilities of the systems further to enable the execution of a broader range of more challenging synthetic methods which, at present, are not possible using the range of automation available in the general synthesis laboratory.

We will describe the use of a solid addition station to facilitate the addition of insoluble reagents, the use of septum piercing hands for reaction set-up and reaction sampling under inert conditions and the ability to execute low temperature chemistry.

These developments have been undertaken against a backdrop of an evolving Visual Basic front-end to the Zymate Easylab Software to allow for a 'walk-up' approach to the technology.

(1) Automated Chemical Synthesis. Brian G. Main and David A. Rudge, p. 425. Proceedings of the International Symposium on Laboratory Automation and Robotics 1994.

(2) The creation of an Integrated Data handling System for Automated Organic Synthesis. C. Green, p. 164. Proceedings of the International Symposium on Laboratory Automation and Robotics 1996.

(3) Development of Workstations for Filtration and Purification of Samples Prepared by Automated Synthesis. D. Rudge and M. Crowther, p. 264. Proceedings of the International Symposium on Laboratory Automation and Robotics 1997.

(4) A Versatile, Fully Automated, Open Access Work-Up Station Designed to Serve the Needs of the Rapidly Expanding Field of Multiple Parallel Synthesis. G. A. Hamlin. Proceedings of the International Symposium on Laboratory Automation and Robotics 1998.

(5) A Compact, Fully-automated Solution-phase Reaction Work-up Facility for Multiple Parallel Synthesis. G. A. Hamlin. Proceedings of the Internationl Symposiium on Laboratory Automation and Robotics 1999.

### cAMP-Screen™: cellular cyclic AMP quantification in an automated 384-well format

*Anthony C. Chiulli, Tropix/Applied Biosystems, Bedford, MA*

The second messenger, 3′,5′-cyclic AMP (cAMP), is a highly regulated molecule that is governed by G protein coupled receptor activation and other cellular processes. Measurement of cAMP levels in cells is widely used as an indicator of receptor function in drug discovery applications. We have developed a non-radioactive ELISA for the accurate quantitation of cAMP levels produced in cell-based assays. This novel competitive assay utilizes chemiluminescent detection that affords both the sensitivity and the dynamic assay range that has not been previously reported with any other assay methodologies. The assay has been automated in 96- and 384-well format, providing assay data that is equivalent, if not better, than data generated by hand. Our data indicates the feasibility of utilizing this assay methodology for monitoring cAMP levels in a wide range of functional cell based assays for high throughput screening.

### Putting sheet screening into practice at Abbott: ramping up a new screening technology

*James Kofron, Lawrence Vernetti, Steven Anderson, Patrick Hymphrey, Carol David, Martin Voorbach, Jennifer Donnelly, Xiaoling Xuei, Yihong Fan, Sujatha Gopalakrishnan, Uha Warrior and David Burns, Abbott Laboratories, Abbott Park, IL*

Abbott's Continuous Format High Throughput Screening (CF-HTS, sheet screening) is a novel, high-density screening platform based on a well-less array (8640) of compounds and gel assay technology. This high throughput screening format has all of the advantages associated with miniaturization (e.g. low reagent consumption, low cost) while providing several advantages over other high-density plate screening (heterogeneous and homogeneous assays; no evaporation, liquid handling, or edge effect issues; minimal automation requirements). Abbott has used this technology for production screening since mid-1998, and is ramping up and extending this technology in pursuit of a goal to run half of our activity-based screens in this format by the end of 2000. The basic technology behind CF-HTS will be presented with several diverse assays as examples, and the modifications to our screening infrastructure to support this effort will be discussed. We will present our current plan to resolve bottlenecks in the areas of data reduction, compound cherry-picking and reformatting, gel manipulation, and imaging systems.

### Laboratory 2000: the challenge of achieving efficiency and compliance

*Alan Potter, GlaxoWellcome, Zebulon, NC*

Significant advances within the field of laboratory automation and instrumentation have greatly benefited the pharmaceutical industry in its quest to discover, develop and monitor the quality of its products. Necessitated by the need for efficiency and greater productivity; faster and more cost-effective means of analyses exist in the form of devices made up of complex electro-mechanical components, all logically controlled and most with the capability to interface with sophisticated information systems. This benefit does come with a price, a greater responsibility to ensure data quality while complying with increased regulatory requirements. Commitment to this responsibility presents a substantial challenge to scientists and managers throughout the industry. Due diligence must be demonstrated. A comprehensive evaluation of every laboratory system utilized, a solid plan of action for correcting any known deficiencies including upgrades or complete replacement, and an accurate monitoring procedure with the ability to measure progress are all absolute necessities to ensure success. Cross-functional term effort and communication must transpire with full managerial support. Vendors need to be audited, made aware of any functional or quality inadequacies they possess as well as the pharmaceutical industry's expectation for these shortcomings to be rapidly corrected. Suppliers of these systems should also be encouraged to provide complete 'off the shelf solutions' to eliminate the need for in house customization. The requirements for regulatory compliance in today's electronic environment have been well publicized. The players involved are not only listening, but also taking the necessary steps to retain and improve efficiency without sacrificing quality. With the proper measures, planning and action; a highly automated, cost effective and compliant laboratory operation can become a reality.

### Building an electronic data/compliance foundation utilizing wireless eClipboards

*Mike Stroz, AstraZeneca Pharmaceuticals, Westborough, MA*

The pharmaceutical industry is rapidly transitioning to flexible, multi-site global organizations operating under demanding regulations. Effective information management provides a foundation for high productivity, rapid response, and regulatory compliance. The industry can no longer rely on today's combination of paper and electronic records. Astra Zeneca has incorporated the VelQuest ePMC™ (electronic Process Management and Compliance) system to address these issues and more. The ePMC™ provides primary data capute at the source creating valid electronic records, and linking these records to the procedures from which the data was created. This electronic platform facilitates compliance with cGMPs and 21CFR Part 11.

### My continuing adventure with 21CFR Part II: the evolution of Zymark's compliance

*Stephen Dobro, Zymark Corporation, Hopkington, MA*

A renewed focus has been given to the three year old regulation, 21CFR Part 11. The speaker will present a chronology of the process of validating a previously developed and commercially available instrument for compliance to 21CFR Part 11. This will include all aspects of the exercise which include familiarization with the standard, development of the protocols, review of the protocols by industry experts, pharmaceutical user company review, execution, exception reports, and any necessary product revisions. Zymark's TPWII rev 2.0 was chronicled because it has been recently developed and reasonably expected to meet the requirements of 21CFR Part 11.

### High throughput enzyme inhibition assay for the quantitative determination of Simvastatin-derived HMG-CoA reductase inhibitors in human plasma using a Tecan genesis 200 robotic workstation combined with 96-well plate technique

*Wei Fang, L. Liu, J.Y-K Hsieh, J. Zhao, J. D. Rogers, B. K. Matuszewski, M. R. Dobrinska, Merck Research Laboratories, Merck & Co., Inc., West Point, PA*

The cholesterol-lowering drug simvastatin (SIMV) reduced heart attacks by 42% in patients who had high cholesterol levels and suffered from heart disease. Upon oral administration, SIMV is quickly hydrolyzed to its β-hydroxyacid (β-HA) and other acid metabolities, which are potent inhibitors of 3-hydroxy-3-methylglutaryl coenzyme A (HMG-CoA) reductase.

Over the last 10 years, Tecan single-probe and Zymark single-tip robots have been used to perform an enzyme inhibition assay for the determination of the concentration of HMG-CoA reductase inhibitors in human plasma. Although both robots do relieve the analyst from performing some tedious, repetitive, and time-consuming tasks, low sample throughput, frequent analyst's intervention, and mechanical instability were observed. This enzyme inhibition assay has recently been modified to achieve higher sample throughput and improve assay reproducibility and mechanical stability by taking advantage of 96-well plate technologies and a Tecan Genesis 200 robotic workstation equipped with 8 fixed tips and some customized hardware and accessories.

The modified assay was validated over the concentration ranges of 0.4 to 20 ng/mL, and 2 to 50 ng/mL for SIMV and β-HA. Intraday precision values (C.V.) for replicate analysis ($n = 5$) of standard curve samples were less than 5 and 9%, respectively. Intraday accuracy ranged from 95 to 105% of nominal value for the two concentration ranges studied. The interday precision of the quality control (QC) samples was less than 2 and 8%, respectively. The respective interday QC accuracy values were 93 to 103% and 97 to 104%. Good linearity across the two concentration ranges was obtained with acceptable reproducibility. This validated assay has been utilized to analyze human plasma samples from several simvastatin clinic studies. Full details of the assay methodology will be presented.

### Robotic system for handling infectious clinical and pre-clinical serum samples

*David Pechter, Robert Firman, Jun Chen, Eric Chaplin, Schering-Plough Research Institute, Union, NJ; Eugene Lochart, The Baker Company, Sanford, ME; Rick Johnstone, Schering-Plough Research Institute, Union, NJ*

The Bioanalytical Laboratory at the Schering-Plough Research Institute is a service laboratory which performs drug level measurements in serum in support of pre-clinical toxicological tests, as well as human clinical trials for anti-viral compounds. A new robot system for the laboratory had two external constraints put on it, besides those typical for a robotic system: since it handled infectious serum samples, it needed to be contained within a biosafety cabinet, and all data handling and labelling were subject to regulatory oversight. The result is a robot cell consisting of a large custom biological safety hood with an integrated track mounted robot, refrigerated storage, mixing, 2-D bar code reading, and automated pipetting. In addition to the material handling hardware, sample labelling software was developed for use at sample collection sites within Schering-Plough, and at external contract laboratories.

### Automated dual LC/MS/MS system doubles the sample throughput of a single mass spectrometer

*Larry S. Birkemo, Stephen A. Wring, Lloyd Frick, Jonathan B. Morgan, Jimmy Bruner, James Ormand, Glaxo Wellcome Inc., Research Triangle Park, NC*

*In vitro* drug discovery screens generate large numbers of samples for analysis and have a pivotal role in accelerating drug discovery. To keep pace with the need for increased bioanalytical capacity, we have developed an automated dual LC/MS/MS system that doubles the sample analysis capacity of a single mass spectrometer.

In a typical LC/MS/MS chromatographic run, the flow path is diverted to waste at the beginning and end of the run, resulting in idle time for the mass spectrometer. We synchronized the injection of samples into two HPLC systems and their flow paths such that when the flow from one system is being diverted to waste, the flow from the other system is being analyzed by the mass spectrometer.

Dual LC/MS/MS was performed using two HP1100 binary pumps, a 6-port switching valve, 2 chromatography columns, and a Gilson 215 autosampler (equipped with 2 Gilson 819 injection ports) interfaced to a PE Sciex API2000 mass spectrometer.

Integrated control of the timing of sample injections, column-switching, and start of the pump gradients was achieved with customized HP Chemstation macros and a Visual Basic™ application. Customized user-input screens were accessed by menu choices added to the standard Chemstation user-interface.

The system has proven to be robust: >5000 chromatograms have been generated without failure. Use of dul LC/MS/MS results in a 2 fold improvement in sample throughput. Combined with other techniques (e.g., cassette analysis or fast gradient chromatography), up to a 16 fold improvement in productivity vs traditional LC/MS/MS has been achieved routinely in our laboratory.

### A validated automated sample preparation for Lc/Ms/Ms to facilitate a multi-step extraction for analytes of differing polarities from plasma

*John R. Kagel[a], Rupinder Bhamra[b] and Christine Swenson[b], [a]Primedica Corporation, Worcester, MA and [b]The Liposome Company, Princeton, NJ*

An LC/MS/MS method, using automated sample preparation for the isolation of potentially unstable drug and multiple metabolities from plasma, was validated and used for sample assays. Due to the polarity range of the analytes, it was not possible to extract each analyte in acceptable recovery by using one step. Instead, one group of analytes with similar polarities was isolated from plasma (Step 1) using a liquid-liquid extraction. The organic layer was removed and evaporated. Next (Step 2) the remaining group of analytes was isolated using a protein precipitation. The organic layer from Step 2 was added to the residue from Step 1 and evaporated. After reconstitution and centrifugation, the supernatant was transferred to injection plates for assay of all analytes in a single run. The differences in extraction conditions also were reflected in comparable differences in optimized LC/MS/MS conditions. Therefore, it was considered advantageous to use separate analog internal standards for analytes from each of the two extraction steps. Automated parallel processing for the multiple transfer steps was used to increase throughput significantly. Summary statistics for the three days of validation will be reported.

### Speeding metabolic stability assays using automated, high throughput LC/MS techniques

*Kelly Johnson*[1], *J. Erve*[2], *A. Dandeneau*[2], *M. Swartz*[1], *B. Kenney*[1], [1]*Waters Corporation: Milford, MA*, [2]*GENTEST Corporation: Woburn, MA*

In drug discovery, metabolic stability is often a key factor in whether or not a compound continues on in the development process. Metabolic stability can be assessed *in vitro* using pooled liver microsomes obtained from humans or other species of interest. To meet the growing demand for rapid analysis of the large number of samples generated by metabolic stability assays, automation has become necessary to increase sample analysis. The use of LC/MS analytical methods can provide the required selectivity, sensitivity, and speed to produce quality data rapidly.

This presentation will demonstrate the use of LC/MS to measure samples produced in a metabolic stability assay. Taking advantage of such factors as parallel sample processing, high sample capacity, and shorter columns to reduce cycle and analysis times, we can analyze the large number of samples produced, and ultimately help expedite the drug discovery process.

### Increasing productivity through a combination of automation and robotics: a case study of assay services

*Melissa L. Cheu, Genentech, Inc., South San Francisco, CA*

The Assay Services Department at Genentech, Inc. is a service laboratory that performs drug level measurement and antibody testing in support of pre-clinical animal studies and human clinical studies. As the number of Genentech products has increased, so have the number of studies, resulting in an annual increase in the number of samples generated and an increased demand for assay support. Assay Services has addressed this by increasing the efficiency and productivity of sample handling and assaying through the automation of various procedures. All sample dilutions are now done by automated dilutors, reducing the number of reassays and virtually eliminating the repetitive stress of manual sample dilutions. In addition, two complete ELISA robot stations have been in use over the last two years that have increased throughput by increasing the number of plates per run (10–15 96-well microtiter plates), and by allowing assays to run overnight without requiring the presence of lab personnel. The net gain from the automation efforts has been to double to number of samples run without increasing the number of lab personnel.

### Automation of an SPE HPLC assay for atenolol levels in human serum

*Joe Long, Gleen Smith, Jimmy Bruner, James Ormand, Glaxo Wellcome Inc., Research Triangle Park, NC*

To increase productivity in the bioanalytical laboratory, we have combined several automated steps, from specimen tube log-in and handling to extraction and HPLC injection, to make the process as hands-free and efficient as possible. These steps combine the advantages of time-saving with safety, allowing less exposure to biohazards and laboratory chemicals.

This poster will discuss the steps in developing an automated method for analysis of Atenolol in human serum. The method was developed to support clinical trials involving a new anti-diabetes drug at Glaxo Wellcome. Atenolol is a selective $\beta_1$-adrenoceptor antagonist used in the treatment of hypertension. Since many people with diabetes are also taking anti-hypertensives, this study was conducted to determine the effects and safety of this combination.

Automated sample handling involves the use of preassigned barcoded specimen tubes of a specific size. Tubes are scanned into a bioanalytical spreadsheet, arranged into racks then placed on tube uncapper/capper instrument. After uncapping, the rack is positioned on the Tecan deck that is part of a combination Zymark/Tecan 96-well sample prep robotic system. When sample extraction is complete, the 96-well plate is placed in an autosampler and run overnight.

Other systems available in our laboratory include one with the capability of transferring small volume samples into 96-well plates, with gravimetric confirmation. Mass spectrometry is also used, allowing shorter run times and more specific compound identification.

### Automated protein precipitation in a 96-well microtiter filter plate

*Beth A. Boman and Robert Pranis, 3M Filtration Products—Empore, Mendota Heights, MN*

Emerging bioanalytical trends are demanding that discovery, metabolism and clinical groups analyse a greater number of compounds in a shorter amount of time. Sample preparation is now the rate-limiting step to achieving higher throughput. Protein precipitation in a 3M Empore® 96-well microtiter filter plate automated on a Beckman Biomek® 2000 Workstation offers rapid sample preparation for the analysis of drugs and metabolites in biological fluids.

The Biomek® 2000 Workstation offers many features that reduce the risk of error and improve productivity. Pipetting, diluting and dispensing operations are performed quickly, easily and automatically. The 3M Empore Filter plate removes precipitated proteins by a 3M patented graded density filter composed of polypropylene. The product has been experimentally determined to retain 98% of all particles larger than 10 μm in size.

The Biomek® 2000 Workstation performed sample transfer and internal standard addition to the samples. The protein precipitation and sample filtration occurred on the filter plate with vacuum from the online vacuum manifold with simultaneous sample collection. The final step to the sample preparation was addition of a mobile phase compatible buffer to make each sample ready to inject. The samples were analyzed via HPLC and the recovery and reproducibility results are reported.

### Determination of drug permeability by filter-immobilized artificial membranes and detection by LC/MS

*Konstantine Tsinman[1], E. H. Block[2], A. Avdeef[1], M. Strafford[1], B. Kenney[2], M. Balogh[2], K. Yu[2], [1]pION, Inc. Woburn, MA, [2]Waters Corporation, Milford, MA*

Prediction of oral obsorprtion properties of lead compounds is a tool that can help discovery teams make the decision of go, no no, or repair prior to candidate selection. A new *in vitro* model of permeability based upon filter-immobilized artificial membranes composed of phospholipids was combined with an LC/MS detection method. Well-characterized compounds and extracts of the kava plant (lactones) were processed through the decribed system and compared with results from detection by UV spectrophotometry. In addition, a cassette experiment of five of the lactones was performed and comparison made to values for the individual compounds.

**Methods**. A series of model compounds and extracts of the kava plant were processed with a pION PSR4p instrument, and their permeabilities were derived from differential UV absorption. The same samples were additionally analyzed using a generic HPLC/MS method. Alternating positive/negative electrospray ionization in the full-scan mode was chosen as the most applicable interface and scan regime. Following data acquisition, the resulting data were processed and a variety of quantitative and qualitative information was obtained.

**Results**. Permeability coefficients based on relative LC/MS peak areas for each analyte compared favorably with those obtained by UV determination. In addition, similar results were obtained even when the compounds were present as a mixture. HPLC/MS detection also allowed for limited identification of unknown components in supposedly 'pure' standards.

### Performance of the AtheNA Multi-Lyte ANA test system; a homogeneous, microparticle-based multiplexed ANA test system

*Mark Kopnitsky, M. Connelly, D. Rran, K. Cichonski, J. Torres, G. Lin, J. Hablawai, M. Desiderio, Zeus Scientific, Inc., Raritan, NJ*

A study was conducted to assess the performance of a commercial, flow cytometry-based microparticle system for the simultaneous detection of thirteen different antinuclear antibody (ANA) specificities. Recombinant or affinity purified proteins (SSA, SSB, Sm, RNP C, RNP 68, RNP A, RNP BB′, Scl-70, Jo-1, Centromere B, and histones) were covalently bound to the surface of distringuishable polystyrene microparticles. Antigen-specific microparticle sets were mixed to form a multiplexed ANA substrate. Patient sera were evaluated for the thirteen different specificities simultaneously in a homogeneous assay format. Sera from 119 normal blood donors were used to evaluate assay specificity and sera from 110 clinically characterized autoimmune patients were used to assess assay sensitivity. ANA HEp-2 indirect fluorescent assay (IFA) was used as the reference method. Individual commercial ELISAs were used to resolve discrepancies. In the normal donor group, concordance to ANA HEp-2 was 87.2%. Of the 15 discrepancies, 11/15 (73%) were negative on the AtheNA assay and positive by IFA. In the autoimmune group, concordance was 89.1%. Of the 12 discrepancies, 8/12 (67%) were positive on the AtheNA assay and negative by IFA. Following the resolution of discrepant specimens, the percent sensitivity, specificity and agreement relative to IFA was found to be 98.8%, 92.9% and 97.3% respectively. We have concluded that the AtheNA Multi-Lyte ANA Test System provides a homogeneous, multiplexed objective means to detect specific ANA with sensitivity and specificity comparable to that of ANA HEp-2 IFA.

### Protein bound drug binding determinations with a 96-well ultrafiltration device for ADME applications

*Aldo M. Pitt, Sara Gutierrez, and Micky Tortorella*, Millipore Corporation, Danvers, MA, *DuPont Pharmaceutical Co. Wilmington, DE*

A critical parameter required as part of ADME screening is the determination of small molecule binding to serum proteins. Traditionally, these measurements have been determined by equilibrium dialysis techniques with ultrafiltration used as an accepted alternative method. Both of these methods have been time consuming and tedious since single devices had to be handled. The purpose of this report is to characterize a new 96-well ultrafiltration device for its low binding properties and also to directly compare the protein binding results achieved to equilibrium dialysis. The use of a new low binding plastic demonstrated increased compound recoveries even when directly compared to polypropylene. A correlation coefficient of 98% was calculated when the equilibrium dialysis and 96-well ultrafiltration binding results were compared. The 96-well ultrafiltration device provides comparable results to equilibrium dialysis with the speed and convenience of the familiar 96-well format for higher throughput ADME screening.

### Evaluation of bias observed with the Zymark MultiDose automated system

*Gale Grant, P. St.-Laurent, C. Briand, A. Dillolo, E. Kwong, J. Visentini, S. McClintock, Merck Frosst Canada & Co., Montreal, Canada*

The effect of the cannula size of the Zymark MultiDose dissolution system on the dissolution rate for two drug formulations was evaluated. A bias towards a higher dissolution rate was observed for results generated on the automated MultiDose system compared to results obtained from the manual dissolution method. The bias was more obvious for the lower strength dosage forms at lower paddle speeds. Several experiments resulted in the conclusion that the bias toward higher results with the automated system was due to the additional disturbance caused by the presence of the sipper cannula during the sampling period. There is a significant difference in the external diameter of the MultiDose cannula (containing thermistor) and those used as USP manual cannula. As well, the cannula on the MultiDose system remains in the media for a longer period of time during sampling due to the maximum allowable filtration flow rate of 12 ml/min.

As a result of this bias, a prototype MultiDose head that consists of two modified stainless steel cannula, one for drawing sample and the other as a thermistor, with similar internal and external diameters to standard cannula used for manual dissolutions was evaluated with the two drug formulations. The data generated with this system was comparable to the manual methods in contrast to data generated with the original MultiDose sipper cannula.

### Streamlining the dissolution test: automated HPLC techniques

*Kelly A. Johnson, Michael E. Swartz and Patricia A. Fowler, and Charles H. Frasier, Waters Corporation, Milford, MA*

The fast pace of the modern pharmaceutical industry requires laboratories to reduce the analytical burden of their test procedures, and increase productivity while still satisfying regulatory compliance. There are now several ways to meet these challenges in the dissolution laboratory. Automated dissolution systems eliminate the slight variations that may occur in manual methods, thereby insuring reproducible data, higher throughput, and cost reduction. Validated single source software control of the entire automated system, as well as dissolution data acquisition and calculations, can further streamline the work flow while maintaining FDA compliance, as with 21 CFR Part 11. As a significant time and resource saver, the USP now approves several methods that involve the analysis of pooled dissolution samples, in which individual dissolution aliquots are pooled and assayed as a single sample. Modern on-line HPLC dissolution systems can pool the individual samples and perform these types of analyses automatically, unattended. Similarly, the ability to automate sampling at shorter intervals and analyse a large number of samples via an on-line HPLC system may provide a more complete solution for the decision making process in the early stages of drug development. Also, these systems must also be capable of handling increasingly complex formulations, such as multiple actives, widely varying dosages, as well as media types being used, such as buffers, organic solvents, and surfactants.

We will present data generated by a single hardware and software source for a number of the above pharmaceutical dissolution applications, summarizing a complete approach to reducing the analytical burden and increasing sample throughput in the dissolution laboratory.

### Development of a TPW II sample preparation method for assay, impurity, CU and D-isomer for Levaquin tablets

*Suman Sharma, Steven Lum and Yolanda Scypinski, Ortho-McNeil Pharmaceutical, Raritan, NJ*

A TPW II method for Levaquin Tablets was developed for the sample preparation of 250 mg, 500 mg and 750 mg tablets. The original manual sample preparation method involves soaking five tablet in the diluent for 30 minutes, shaking for an additional 30 minutes followed by settling the sample solution for one hour. A dilution step is required after settling. Using the new TPW II method has reduced the sample preparation time by half and has increased the accuracy and precision of the method.

This poster primarily discusses the TPW-II method development stages, the problems encountered and the resolutions to the problems.

A new consolidated method was also developed and used for the analysis of all the above tests for this work. The current chromatographic procedure uses one method to monitor potency, impurity and CU while a second method is used for the D-isomer.

### Automation of the analysis of fluorides in toothpaste, using a Zymate XP robot system

*Caroline J. Tillett and Teck M. Khong, SmithKline Beecham Consumer Healthcare, Weybridge, Surrey, UK*

A robotic system, for the analysis of fluorides in toothpaste, has been custom designed by Zymark to SmithKline Beecham Consumer Healthcare (SBCH) specifications. The analysis of fluorides within SBCH, is a time consuming, repetitive operation. The automation of the methodology has been shown to deliver several benefits:

- Greater throughput in processing fluoride analyses.
- Increase in quality and reproducibility of fluoride analyses.
- The system is fully validated and the results are posted to LIMS directly, with full audit trail.
- Redeployment of resources for more effective and better usage.
- Reduction in analysis time by a factor of 4.5.
- 50% decrease in the cost of consumables.

Different methods are performed for fluoride analysis, due to varying sample matrices and varying sources of fluorides and different requirements of individual countries. The robot has been designed to handle all the different methods presently in use, as well as any new methods that may be introduced. The robot prepares samples by the specified method, then analyzes by ion selective electrode (ISE). The results are collated in a report, which can be printed out or can be directly posted to LIMS *via* a LIMSlink.

The robot has also been designed to perform the calibration of the electrode. A common problem with ISE is drift of the electrode. The robot will analyze the drift by measurement of a 1 ppm standard in between samples, if a drift of greater than 2% occurs the robot will calibrate the meter, then continue reading the samples.

### Automation and marketed products: a method development case study

*Christopher A. Zordan, David K. Lloyd; DuPont Pharmaceuticals Co., Experimental Station, Wilmington, DE*

Developing automated sample preparation methods for marketed products raises issues not usually encounted during 'first-look' method development activities. Marketed products have established analytical histories and methods. Having this knowledge allows for some shortcuts, such as removing the need to develop and test dilution solvents and HPLC parameters. On the other hand, automation techniques can not exactly replicate manual preparation methods, which may lead to extra validation work to prove equivalence.

Coumadin® (Warfarin Sodium, USP) is a marketed product within an established history of analytical methods. An automated content uniformity method was developed from an existing manual method and an existing automated composite assay method. The following points will be examined: 1) the decision to use existing HPLC parameters to limit development work to the sample preparation, 2) the validation of a new standard linear response range of 0.02–0.1 mg/mL due to a combination of dosage strength and instrument limitations tht lead to low maximum concentrations for the lowest dosage strengths, and 3) lowering the internal standard concentration to 0.05 mg/mL so that it lies near the middle of the new linear range of the standard.

### MultiDose™ dissolution testing of a moisture sensitive product using modified carousel caps to control localized humidity in environment surrounding tablets staged for testing

*Joseph Dremock, G. Martin, D. Reed, P. Harmon, L. Magiso, L. Fegely, Merck & Co., West Point, PA*

An automated dissolution method using the Zymark MultiDose™/MultiFill™ was demonstrated to be equivalent to the manual procedure for an immediate release drug product that has known sensitivity to moisture. During development of the automated procedure it was determined that exposing the tablets to the typical moisture encountered while in the carousel above the dissolution vessel prior to testing caused the dissolution rate of the tablets to increase at the early time points (10, 15 and 20 min.). In order to eliminate this effect, carousel caps were modified to include a canister of desiccant suspended in each of the 8 wells of the carousel while the tablets were awaiting testing. When the results of tablets tested in this way were compared to tablets tested manually, the mean results were within the specified acceptance criteria.

### Automated sample preparation using a TPWII compared to a semi-automated stand alone extractor

*Ron Muenz, Maria Styslo-Zalasik, John Troisi, Ronnie McDowell and Stephen Scypinski, Analytical Chemistry Research and Development, The R.W. Johnson Pharmaceutical Research Institute, Raritan, NJ*

A fully automated and a semi-automated sample preparation procedure was developed using a five tablet composite for HPLC analysis. The automated method uses a Zymark TPWII® robot, while the semi-automated method uses a Stand Along Extractor® from Source for Automation. Each technique uses homogenization for tablet disintegration. Both methods were validated and shown to be equivalent. The two methods are an improvement over the existing manual method which uses sonication to disintegrate the tablet. This poster will discuss the advantages and disadvantages of each technique.

### Opening the door to a 'Paperless' laboratory

*Ed Halpin, VelQuest Corporation, Hopkinton, MA*

Ever increasing regulatory demnds force companies to re-allocate resources from their primary mission to compliance related activities. Market research indicates that up to 70% of laboratory and QA resources are now devoted to compliance. To ensure compliance, the pharmaceutical industry and its partners continue to rely on paper-based systems to achieve the required security and audit trails. Since manual procedures cannot be fully 'validated', GMPs require independent checking and approval of resulting data.

VelQuest's Electronic Process Management and Compliance (ePMC) provides a fully validated 'paperless' approach to acquire all data at its source, store and retrieve this data in a fully secure database, and use this information for new product submissions, product releases, quality assurance and management analysis.

This poster will compare today's manual 'paper-based' laboratory with the 'paperless' laboratory enabled by VelQuest's ePMC technology.

### Efficient data management for accelerated compound profiling

*David S. Williams, Nugenesis Corporation, Westborough, MA*

New technologies are producing an ocean of data, but acting on that information is more than a few steps behind. Certaintly, scientific information has value, but having a lot of data does not mean it is worth a lot.

Within the pharmaceutical industry alone, investments in data generation technologies (high throughput screening, combinatorial and parallel synthesis) have resulted in an unintended consequence—decreased value of data through information overload.

In theory, electronic record keeping is easy. In practice, advanced technologies and lab applications rapidly generate a torrent of bytes—all products of proprietary sources—each stored in its own proprietary 'language,' none able to communicate with another. In the ultimate irony of laboratory automation, the output for each sophisticated instrument is a printed report. Converting data into usable information requires manually piecing together a puzzle of paper.

Nugenesis Corporation has developed a program that systematizes the collection, storage and sharing of scientific data by unifying it into a common electronic format. Scientists can capture data from a variety of lab and business application sources and store it in a central database, while maintaining its graphic integrity. Scientists anywhere in the organization can view the assembled data using a web browser.

### Automated moisture system

*Steven J. Salata, Nabisco, Inc., East Hanover, NJ*

Our laboratory's (Nabisco Research's Analytical Chemistry), major responsibility is testing food products in various stages of development and stored under various conditions to determine shelf life. Moisture content is one of the primary chemical parameters measured. There are a wide variety of methods for the determination of moisture in food and the method selected depends on the type of food being analyzed. The technique we selected to automate is for the determination of moisture based on oven drying. The method employs a convection vacuum oven. Our most common matrix, cookies and crackers are run at 70 °C under vacuum <50 mm Hg (6.7 kPa), for 18 hours. The weight change in the sample is reported as moisture loss. This technique was an ideal candidate for complete automation since we analyze over 5,000 samples a year. Also the method has well defined and simple steps, allowing for easy implementation as an automated analytical process. The majority of products run by this technique are cookies and crackers, which are pourable and require little manipulation.

### Beyond microtiter plates: molecular synthesis and screening in arrays of microchannels

*Colin Brenan[1,2], Tanya Kanigan[1,2], Robert Hess[1] and Ian W. Hunter[2], [1]Advanced Instrumentation Systems, Inc., Lincoln, MA, [2]Department of Mechanical Engineering, MIT, Cambridge, MA*

In recent years, biochemical and pharmaceutical researchers have begun migrating from screening in 96-well plates to screening in 384-well or even 1536-well plates. Screening in smaller volumes conserves space, assay reagents, compound library reagents, and scarce target materials. However, further reduction in well volumes leads to the trapping of gas bubbles, rapid sample evaporation, lower signal levels, and presents a formidable challenge for conventional liquid handling techniques such as pipetting.

In response we have created a novel 'nanotiter' plate technology with the potential to initiate and monitor $10^5$–$10^6$ biochemical assays in parallel with ~100 nl total volume per assay. Our approach purposefully exploits the same micro-scale phenomena that cause problems when conventional microtiter plate technology is miniaturized without innovation in system design.

Assays are performed in uniform addressable arrays of microchannels machined through flat plates. The through-hole dimensions are typically 200–300 μm across and at least 500 μm in length such that each channel typically holds between 25–100 nl of fluid. At these small dimensions, fluid can be transferred into the channels from either face and retained entirely by capillary forces. Inter-channel cross-talk is prevented through application of an exterior hydrophobic coating to the hydrophilic plate material. Stacking (in registration) of multiple microchannel plates rapidly mixes fluids in co-registered microchannels within seconds through a combination of convection and diffusion. Once initiated, all microassays are monitored simultaneously by a low cost fluorescence imaging system based upon a cooled megapixel CCD camera.

In addition to assays used in ultra high throughput screening of small molecule libraries, we envision that this technology platform will be used to perform many other types of sub-microliter chemical or biochemical reactions in parallel. Examples of these other applications include combinatorial library synthesis and diagnostic immunoassays.

### Automating the determination of aqueous drug solubility using laser nephelometry in microtitre plates

*David Proudlock, Malcolm Willson and Barbara Carey, Glaxo Wellcome R&D. Medicines Research Centre, Stevenage, UK*

The ability to determine solubility of candidate drugs in aqueous solution provides valuable information in the drug development process. Nephelometry has successfully been shown to determine this.[1] This poster outlines the implementation of a 'Solubility QC Workstation'. The workstation consists of the Zymark Twister, Lasystem's Multidrop and BMG's Nephelostar. The equipment was integrated using the 'Scrippy' scripting software. The validation work demonstrated good agreement with the manual study.[1] The system has been successfully transferred to the business for solubility determination of IC50 samples.

[1] Bevan, C. D., Lloyd, R. S., *Analytical Chemistry*, **72**, 2000, 1781–1787.

### Process improvements: increasing the throughput and efficiency of the liquid handling group at Glaxo Wellcome

*Jimmy Ballinger, Heather Judy, Brandy Lloyd and Donald Lyerly, Glaxo Wellcome Inc., Research Triangle Park, NC*

High Throughput Screening was introduced at Glaxo-Wellcome RTP in 1996 and was initially supported by the Compound Services Department. In 1997, increases in the demand for liquid samples required the creation of a Liquid Handling Group within the department. Requests were filled using stand-alone automation and software tools based on 96-well format. As screening technology and capacity improved, new processes and formats were requested by R&D scientists. The formats included assay-ready or 'just in time' plates from customized dilutions, 384-well formats, pooling, column daughters for serial dilutions, and creation of retest plates (cherry picks). An INternational Compound Acquistion system (INCA) was also introduced making our compounds viewable and requestable by GW sites worldwide. Due to the addition of these new work practices and the creation of a QC department to enforce the integrity of the liquid samples, scientists became more confident with the liquid stores as shown by a 75% increase in liquid requests over the last year and a half. The increase has led us to evaluate the current processes and tools employed to ensure they would be sufficient to meet our future needs. A Compound Services Design Team was initiated in 1Q 2000 aimed at evaluating these needs. The team kept in mind that we would be working with limited resources, no additional headcount, and a flat budget. Our poster will describe process improvements we have made or plan to make in order to increase the group's throughput and efficiency.

### The step before uHTS: assay miniaturization using automated nL dispensing benchtop equipment

*M. Busch, S. Marose, T. Mander, EVOTEC BioSystems AG, Hamburg, Germany*

The benefits of uHTS (screening in assay volumes $<5\,\mu$L) are well established: faster throughput and lower reagents consumption. However, surface/volume ratios can often increase by the factor up to 100 when applying miniaturized fluid handling. Hence, adsorption of biochemical reagents becomes a major issue in the performance of a miniaturized assay. Thus, special considerations are needed to screen in the 1 μL/well range. For instance the material of the carrier and the dispensing systems have to be considered carefully. Detergent screens or the altering of the sequence of the addition of the reagents can help to overcome the adsorption issues during assay miniaturization. Several worked examples based upon data including receptor-binding, vesicle based assays, DNA-protein interaction and kinase/phosphatase assays will be presented. Our results show that most of the assays require specific additives for a sufficient performance in the micro scale. A general procedure for performing detergent screens in an iterative mode will be discussed.

### Understanding liquid handling on a Packard Multiprobe® II

*James Ormand, Jimmy Bruner and Larry Birkemo, Glaxo Wellcome Inc., Research Triangle Park, NC*

Experimental design is a tool that can be applied to gain an understanding of many laboratory processes such as reaction, assay, and instrument optimization. Our automation team primarily uses experimental design to optimize instrumentation such as liquid handling platforms. Liquid handlers allow the user to optimize for specific types of assays by offering up to 12 different parameters for controlling aspiration and dispensing. Determination of which parameters are important for a particular type of liquid can be a time consuming task.

In 1998, we reported on the use of experimental design to improve the liquid handling of organic solvents on a Tecan Genesis. This poster will compare our findings on the Tecan Genesis with the results from a current study of liquid handling on a Packard MultiProbe® II. The current study will also include examination of several additional factors including pipet tips.

### CPC microreaction technology—innovative tools to improve the quality of organic chemistry and to accelerate R&D processes in the life science industry

*Shahriyar Taghavi-Moghadam and Axel Kleemann, CPC Cellular Process Chemistry GmbH, Frankfurt, Germany*

The first microreaction system now has become commercially available; this innovative system provides scientists with an advanced technology for improving and optimizing the synthetic procedures in the chemistry. The precise control of the reaction parameters such as temperature, reagent concentration gradient allows an explicit managing of the reaction proceeding for higher selectivity and increasing of the reaction yield.

By using a CPC modular microreaction system, a continuous process instead of batch processing, with higher quality of reactions as a consequence of the unique features of microreaction devices, chemists would benefit from the flexibility in their day-to-day work. The vastly reduced hold-up of dangerous reactants allows an easy and safe handling of reactions with potentially hazards.

In addition, the involved time and investment would be significantly reduced. The same developed process for small amounts of compounds in microreactors can be used to synthesize kg- or even ton-samples in parallelized arrays. This will lead to a higher R&D throughput and thus to more drug candidates in the R&D pipeline.

Additionally to design and construction of microreactors CPC focuses on the application of organic chemistry in modular microreaction devices and will demonstrate the feasibility and importance of this excisting new technology for the life science and the fine chemical industry.

### 384 well small volume plates vs. 1536 well plates: two platforms for the 2 to 10 μL range

*Rainer Heller*[1], *Anja van der Ploeg*[1], *Johanna Neumayer*[2] *and Guenther Knebel*[1], [1]*Greiner Labortechnik GmbH, Frickenhausen, Germany*, [2]*Tecan Austria GmbH, Groedig, Austria*

The progress in automation and assay detection are the driving forces to run assays faster and in smaller volumes. The assay volume proven to be used in further miniaturization will be in the 5 to 10 μL range.

We have investigated other user-friendly plate formats than 1536 well platforms that offer similar savings in reagent consumption, but with faster set-up times at even lower investment. Finally, the well design of a standard 384 well plate has been completely re-modeled and optimized for smaller well volumes and better detection efficiency. Our novel 384 well small volume plate has round wells with a conical well shape and a total well volume of 30 μL (working volume between 5 and 20 μL). These plates enable to perform homogeneous, heterogeneous, and cell based assays. Additional features, like raised rims per well have have added to prevent any kind of cross-contamination.

The data presented will show that both plates have the potential to miniaturize assays in the sub-10 μL range at the same performance. Homogeneous and cell-based assays have been performed using CCD-imaging systems and PMT-plate readers as detection platforms.

### Disposable 96 well plastic microfluidic devices for HTS and DNA applications

*A. Gerlach*[1], *G. Knebel*[1], *A. Guber*[2], *M. Heckele*[2], *D. Herrmann*[2], *A. Muslija*[2] *and T. Schaller*[3], [1]*Greiner Labortechnik GmbH, Frickenhausen (Germany)*, [2]*Forschungszentrum Karlsruhe, Institut für Mikrostrukturtechnik, Karlsruhe (Germany)*, [3]*Forschungszentrum Karlsruh, Hauptabteilung VersuchstechnikKarlsruhe (Germany)*

Microfluidic devices fabricated by mass production offer a wide potential of applications such as high-throughput drug screening, clinical diagnostics and gene analysis. The low unit production costs of plastic substrates make it possible to produce single-use devices.

In close collaboration, Greiner Labortechnik and Forschungszentrum Karlsruhe have developed a single-use plastic microfluidic device in the standardized microplate footprint. Feasibility studies have shown that hot embossing with a mechanical micromachined moldng tool is the appropriate technology for low cost mass fabrication. A subsequent sealing of the microchannels allows submicroliter sample volumes in 96-channel multiplexed microstructures. The microfluidic lab-on-chip structures are compatible with existing plate and liquid handling robotics.

This poster will show a low cost production of 96-channel plastic microfluidic devices to demonstrate the application of microtechnical fabrication processes for high-throughput screening, CE-separation and DNA analysis.

### Glass bottom microplates (96, 384, 1536 well) for high-resolution imaging and single molecule detection

*Andreas Gerlach*[1], *Claudia Kaiser*[1], *Henning Vollert*[2], *Angelika Bastl*[2] *and Günther Knebel*[1], [1] *Greiner Labortechnik GmbH, Frickenhausen, Germany*, [2]*Aventis Pharma GmbH, HTS, Assay Development, Frankfurt, Germany*

Griner Labortechnik and Aventis Pharma (formerly Hoechst Marion Roussel) have co-developed a full set of unique glass bottom microplates (96, 384, 1536 well). The glass bottom plates incorporate high quality optical glass with a thickness of 150 μm bonded to the parent plate. All plates comply to the standardized microplate footprint and provide superior quality in applications where low autofluorescence and optical clarity are required. At the time, these plates are available in opaque black for high-resolution imaging, sensitive fluorescence and confocal microscopy applications, like FCS and single molecule detection.

This poster will focus on the impact of the most relevant features of glass bottom plates vs. plastic clear bottom plates and will demonstrate the superior performance in selected applications.

### Ultra-fast dispensing for HTS in high-density plates

*Don Rose and Tom Tisone, Cartesian Technologies, Inc., Irvine, CA*

As 1536 becomes more widely used for high throughput screening (HTS) applications, there is a need for practical, automated liquid handling solutions for assay assembly. Assay assembly involves the delivery of a series of reagents, typically substrates or protein targets, to 1536 well plates. Addition using a 96-well pipetting station is extremely slow. Each of 16 transfers requires several minutes, which includes an aspirate/dispense to mix the reagents as well as cleaning or disposal of tips. This can lead to a total plate filling time of as much as 30 minutes.

Non-contact ink-jet type dispensing can deliver nanoliter volumes of liquid to high density plates. Cartesian's patented nQUAD™ technology combines the high-resolution displacement capabilities of syringe pump with the high-speed actuating capabilities of a microsolenoid valve to deliver nanoliter volumes in a non contact manner to 384, 1536, 3456 well plates.

A major advancement in this area has been the development of the proprietary synQUAD™ technology which does rapid, 'on-the-fly' dispensing to the plate, much like a large volume inkjet printer. This is accomplished by synchronizing the syringe pump, microsolenoid valve, and the XYZ positioning system. The advantages of the synQUAD technology are:

- **Speed**. A 1543 well plate can be filled with 4 μL per well in **under 30 seconds**. This is up to 60 times faster that a conventional 96 channel pippettor.
- **Efficient Mixing**. The velocity of the liquid being dispensed is produces efficient mixing of reagents in the well.

- **Multiple Reagents**. The channels of the dispenser are independently controlled to allow multiple reagents to be added rapidly to the plate.
- **No Tip Washing**. The non-contact dispense mechanism results in the tips dispensing above the plate such that the tips never touch the contents of the well.
- **Multimode Dispensing**. Reagents can be delivered by either aspirating from a source or bulk dispensing from a reservoir.
- **Dynamic Range**. Volumes from 20 nL to 20 μL can be dispensed.
- **Precision**. Reproducibility of the dispense is on the order of 2% CV in the microliter range and <10% CV in the nanoliter range.

This paper will give an overview of the nQUAD, synQUAD technologies as well as presaent data on linearity, accuracy, and precision for these different liquid handling modalities.

### Miniaturization technologies for high throughput biology

*Peter Coassin, Aurora BioSciences, San Diego, CA*

This talk will summarize Aurora's strategy to integrate Biology with miniaturized automation to enable high throughput screening and biological research. Specific components of the process will be described, including microfluidics, detection, sample handling, and bioassay technology amenable to high density formats. Results from ultra-high throughput (e.g. >100K samples/day) assays will be presented. The implications of the increased productivity these systems allow for determining the functions of genes will be discussed.

### The integration of Tecan Gemini software with a generic machine interface for cherrypicking in the sample supply process

*Andrew Bonner, Lee Chambers, Seb Carey and Neil Benn, Glaxo-Wellcome R&D, Medicine Research Centre, Stevenage, UK*

The liquid handling robots (LHR) used at GlaxoWellcome's Stevenage site for the supply of samples for screening have been integrated with a generic machine interface. The advantages of using a common interface which links the LHRs to corporate sample ordering system ensures high data integrity and reduces process and data errors. Operators manage requests by directing them to particular LHRs, and these are processed using the generic machine interface.

Within the suite of LHRs, there are a number of Tecan Genesis RSP systems which are used to cherrypick discrete samples for secondary screening and projects. There are requirements for greater flexibility and sub-5ul dispensing which can be met by using Tecan Gemini software.

The integration of Gemini software can be achieved by using an ActiveX driver to pull data, for a selected order, from the corporate database, via the generic interface. This data can be converted by the driver into a format that Gemini can use and piped into Gemini via a named pipe. Both the method and worktable for the processing of any order are dynamic and constantly changing as data is passed between the generic interface and the ActiveX driver, and between the ActiveX driver and Gemini.

### A flexible approach to automated compound storage software systems

*David Harding, Patrick Laffargue, David Shimmin and Michael Pollard, RTS Thurnall plc, Manchester, UK*

The pressure of increasing demand for compounds from HTS users and increasing library sizes has caused many pharmaceutical companies to invest in automated compound storage systems. However, while the main aim is common, each company has its own requirements. For example, not only do compound library sizes vary, but so do screening strategies, storage and distribution strategies. In addition there are some very real constraints such as physical space and existing IT systems.

### Automated extraction of natural products from plant tissues

*Bruce E. Richter, Richard E. Carlson and John L. Ezzell, Dionex Corporation, Salt Lake City, UT*

While high throughput screening (HTS) has been applied to thousands of compounds produced as part of combinatorial libraries, there is interest in applying HTS to the pharmacologically active compounds found in plant tissues. However, these 'marker' compounds must first be extracted from the tissues in which they naturally occur before HTS. The extraction techniques normally used to remove the marker compounds from plant tissues require long periods of time and copious amounts of solvents. In addition, none of these extraction procedures can be automated. Accelerated solvent extraction (ASE) has been proven to be effective in removing marker compounds from a variety of plant tissues.

Using ASE, a fully automated extraction workstation, the extraction of marker compounds from medicinal plants is completed in about 15 minutes using only 20 to 30 mL of solvent. Furthermore, ASE may also be used to determine pesticides levels in medicinal plants before processing and packaging. ASE yields of the selected marker compounds have been shown to be equivalent to or higher than other methods for the same marker compounds, while at the same time offering significant economy in time, labor, and solvent use. This system can extract up to 24 samples without any user intervention.

### Assay development in high density MicroWell® plates: use of well geometries, format, surface modification and optical properties to achieve optimal assay performance

*Barbara M. Sullivan, Life Science Discovery Products, Nalge Nunc International, Naperville, IL*

When developing assays in high density, low volume MicroWell plates, it is important to understand how plate material, surface properties, surface to volume ratio and optical properties affect assay performance. Handling issues such as evaporation control, format compatibility with automation, and determining appropriate reagent concentrations and cell densities resurface as basic development points. This presentation is designed to furnish answers to questions such as: 'Is there a perfect surface?' 'Do I even need to consider surface charge in a homogeneous assay?' and 'How much can I reduce reagents and expect reasonable signal to noise ratio?' and to stimulate a discussion regarding practical issues surrounding assay development.

Information to be discussed was developed in the Research and Development labs at Nalge Nunc International (NNI) as well as in collaboration with other academic and industrial laboratories. We examined many parameters comparing 96 well plates to 384 well plates as well as different varities of 384 well plates. Of note:

- influence of plate material and surface properties on assay performance, reagent compatibility and chemical resistance
- comparison of round and rounded square well geometries and effects on liquid cross contamination
- the influence of surface to volume ratios on the reduction of reagents, particularly in assays utilizing modified surface properties
- measurement of cell viability and proliferation
- optimal choice of solid and optical bottom plates as well as the utilization of pigmented black and white plates for radioactive, luminescent and fluorescent assays
- control of mechanical processes, timing the addition of reagents, and the control of evaporation to optimize the use of high density plate in high throughput assays

Typically made of polystyrene or polypropylene, MicroWell assay plates usually consist of a defined array of round or squared wells. We compared the effect and performance of round and rounded square well geometries on typical assays. Square well plates often had problems with cross contamination, whereby liquid from one well would flow to adjacent wells by capillary action. Rounded square wells, in conjunction with the plate material utilized, eliminate capillary action and consequential cross contamination.

Surface modification such as cell culture treatment and high binding surfaces offer distinct advantages for specific assays capable of utilizing the solid phase. The two-fold higher surface to volume ratio in a 384 well plate compared to a 96 well plate results in significantly higher signals for assays utilizing surface properties.

Consequently, reagents can be reduced at a ratio greater than 1:4 while still providing signals comparable to those achieved in a 96 well plate.

With regard to optical properties, solid black and solid white as well as back or white optical bottom plates (OBP) were compared by measuring fluorescent and reflective properties, signal to noise ratios, light cross talk and sensitivity. White plates had greatest reflectivity and lowest background noise particularly for radioactive and lumininescent assays. Black optical bottom plates were superior for fluorescent assays when utilized with bottom reading instruments, showing the lowest background signal and light scatter, resulting in the highest signal to noise ratio.

Other considerations are also important for optimal performance in high density plates. Mechanical processes, dispensing, reading and timing as well as control of evaporation have to be controlled in order to minimize assay variability.

### Second generation, HE™ (high efficiency) microplates in 96 and 384 formats: low volume assays aren't just for 1536 anymore

*Amer El-Hage, Jon Petersen, Robert Danielzadeh, Meng Zhang, Doug Modlin and Mike Biros, LJL Biosystems, Sunnyvale, CA*

Traditionally, there are two primary advantages to migrating to 1536 well formats: assay volume reduction and increased throughput compared to lower density microplate formats. LJL has developed a second-generation HE™ microplate in the 96 and 384 well format that provides similar reagent, target and compound savings compared to the 1536 well format. HE microplates, when used in conjunction with SmartOptics™ enabled instruments such as Analyst™, deliver similar sensitivity and signal to background across a broad range of assay volumes (4 to 40 microliters). This enables researchers to develop assays in volumes amenable to hand-pipetting and then scale down with automated pipetting—all without changing instruments or plates. Several assays will be demonstrated with this microplate, including Serine/Threonine Kinase and cAMP monitoring in a homogeneous HEFP™ format. The HE microplate design, fabrication and quality control methods provide a low auto-fluorescence background, tight plate-to-plate uniformity, and lot-to-lot consistency. These attributes are essential for low volume assay scalability and reliable HTS automation.

### Solving the MALDI-TOF-MS automation puzzle

*Claus Köster, Bruker-Daltonik GmbH, Bremen, Germany*

In recent years matrix-assisted laser desorption/ionization time-of-flight mass spectrometry (MALDI-TOF-MS) has grown rapidly in its importance in the areas of Genom and Proteom research. The method is based on the laser desorption/ionization of a crystallized mixture of analyte/matrix (MALDI) by a short laser pulse. The ions are accelerated inside an electrical field to give all ions the same kinetic energy, which is equal to _ $m/z\ v^2$, where m/z is the mass-to-charge ratio and v the ion velocity. By measuring the time-of-flight (TOF) between the laser pulse and the event when the ions hit the detector, m/z can be calculated. The received average mass spectrum has a high information content and can be acquired in a few seconds. Another reason for doing MALDI-TOF-MS is the possibility of fully automated processing.

But, how to solve the puzzle of creating fully automated a mass spectrum from a real sample?

(1) Deliver the sample in a microwell plate format.
(2) Do parallel processing (8 needles or more, MAP II/8).
(3) Be flexible and use Excel and it's macrolanguage Visual Basic to run the liquid handler.
(4) Use magnetic bead clean up.
(5) Reduce your sample volumes to sub-microliter amount but not lower.
(6) Prepare the sample on a microwell plate MALDI-target (SCOUT MTP).
(7) Use the anchorchip technology.
(8) Use fuzzy-logic and/or visualization software to create a MADLI-TOF-MS.
(9) Use a non-gritted TOF-MS to increase sensitivity and non-disturbed sample picture.

**Confluent sample analysis for ultra high-throughput screening**

*Kerry G. Oliver, Luminex Corporation, Austin, TX*

Multi-Analyte Profiling using suspension arrays is a well established method for increasing throughput while decreasing costs and sample size. The attendee will now learn how to use the Luminex technology to perform ultra high-throughput screening for enzymatic, antigen-antibody, and nucleic acid based applications. The inexpensive system is capable of analyzing 32,000 microtiter wells per day. Multiplexing just 10 analytes per well, the total throughput of a single system is over 300,000 analyses per day. Sensitivity and precision will be demonstrated which equals or surpasses any current system. Techniques for working with kinases, single nucleotide polymorphisms, and expression analysis will be presented.

**Automated massively parallel high-throughput screening using a unique SPE 'Card'**

*Robert Pranis and Jason Jacobson, 3M Filtration Products-Empore, Mendota Heights, MN, John Janiszewski, Mark Cole, Pfizer, Groton, CT Tom Astle, Tomtec, Hamden, CT*

The explosion of new drug synthesis via combinatorial chemistry has generated incredible pressures on the analytical chemist to evaluate and analyze tens of thousands of new compounds a year via *in vitro* tests coupled with SPE and LC/MS. This has led to novel methods for coping with the large numbers of samples. A joint venture between Pfizer, 3M and Tomtec has produced such a method to rapidly screen samples using Empore technology in a unique 'card' format compatible with existing automation equipment.

The Empore SPE Card is a high-throughput screening disposable used to shorten the lead drug candidate selection process by providing parallel chromatography and direct injection into a LC/MS system from the card. The card has the footprint of a 96 well micro-titer plate and has 96 elution zones. Automated activation, loading and washing steps were accomplished utilizing a modified Tomtec Cell Harvester. Direct injection of the drugs into the mass spectrometer was accomplished by elution utilizing a prototype device. Versatile, easy-to-use software provides powerful synthesis control along with extreme simplicity.

**High-speed 96-well format oligonucleotide synthesizer**

*Damien Luk, Robert D. Guettler, Jimmy Koh, Anh Tuyet-Doan and Aaron Schohn. GeneMachines, San Carlos, CA*

Oligonucleotides are essential elements of genomic research. The PolyPlex™ oligonucleotide synthesizer makes 96 different oligos in standard 96-well format, which is amenable to downstream high-throughput processing and handling. Operator time is minimal and synthesis time is less than 3 hours for a 96-well plate of 20-mers. By eliminating flushing of reagent lines through a parallel dispensing technology, optimal synthesis time and reagent consumption are achieved. PolyPlex's low reagent consumption generates oligos quickly, at low costs and small scales as low as 10 nmol. Synthesis costs, including all consumables, are less than $0.10 per base. The PolyPlex synthesis chamber provides an inert-gas environment where synthesis progress can be monitored by using full-plate trityl collection after any base addition. PolyPlex utilizes fully licensed chemistry and generates oligos with greater than 98% coupling efficiency. Versatile, easy-to-use software provides powerful synthesis control along with extreme simplicity.

**Micro-centrifugation for small volume nucleic acid sample preparation: an effective, cost-saving technology**

*Nancy Bergsteinsson, Ben Riepe, Anthony Matero, Tad Finkler, Melinda Au, Poonam S. Medberry and Carl U. Buice. GeneMachines®, San Carlos, CA*

The newest generation of capillary sequencers has challenged the capacity of current sample preparation technologies. As researchers prepare millions of samples for squencing, they are required to prepare large numbers of samples, and are faced with steep costs. We have developed and tested a new centrifugation technology that hopes to address these problems in sample preparation.

The GeneMachines® Array Centrifuge is a novel approach to centrifugation, to any preparation technique requiring separation. It arrays 96 individual rotors, each a vessel for both centrifugration and sample processing, which spin up to 14,000 g. The integration of this unique centrifuge into a liquid-handling platform creates our RevPrep™ workstation for automating standard protocols, in an unattended, high-throughput manner. The rotors enable drastically decreased centrifugation times, increased throughput and reduced costs.

We will show how the Array Centrifuge impacts various protocols, including plasmic preps, BAC preps and PCR product clean-up, by explaining the technology and results from the RevPrep™.

### High throughput synthesis, deprotection, purification and analysis of ribozymes

*Mark Sanseverino, Lara Maloney, Laurent Bellon, Kuyler Jones, Bryce Keen, Victor Mokler and Leonid Beigelman, Ribozyme Pharmaceuticals, Boulder, CO*

Cell culture-based primary screens of a large number of ribozymes is a critical component of the accelerated drug discovery program at RPI. In order to produce the necessary ribozymes in a high throughput manner RPI has reformatted and automated the olignucleotide production process to a 96-well-based format. This poster exemplifies the solutions selected for each of the four integral components of the ribozyme production process; Synthesis, Deprotection, Purification and Quality Control/Analysis.

The 96-well format in tandem with the workstation platform allows a rapid and well-controlled production of ribozymes. This production process has the capacity to produce 2000 oligonucleotides per month under its current configuration.

### Evaluation of 384 kinetic assays as a screening platform

*Christine Ladislaw, Cameron Stuver and Mark Namchuk, Vertex Pharmaceuticals, Cambridge, MA*

The use of data obtained from one drug development program to gain an advantage in a subsequent project is a longstanding goal of the pharmaceutical industry. This concept is best illustrated when families of related targets are being pursued (kinases, GPCR's, etc.) where the ability to predict selectivity issues early on has tremendous practical benefit. It is also a centerpiece for the Vertex Chemogenomics initiative. In order to generate large quantities of HTS data of sufficient quality for this type of analysis, we have developed a 384 kinetic enzyme assay platform for kinases using a standard coupled enzyme assay. Automation consists of standard Zymark robotics platform upgraded with a pair of Hypertask quadrant positioners. Development issues particular to small volume kinetic assays are discussed. One of the clear benefits of screening kinetically is low false positive rates. Approximately 70% of the compounds we observe as hits, with this method provide demonstrable dose responses when retested with fresh samples of the compounds. Other benefits are discussed below.

### An efficient and fast procedure for the Hantzsch dihydropyridine syntheses using microwave based coherent synthesis™

*Jacob Westman[a] and Liselotte Öhberg[b], [a]Uppsala, Sweden, Personal Chemistry AB, [b]Uppsala, Sweden*

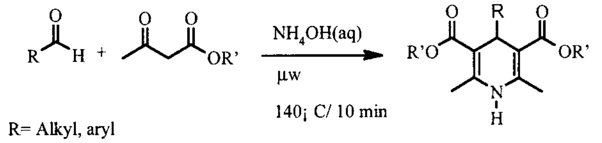

The preparation of 1,4-dihydropyridines by classical Hantzsch synthesis,[1] a one-pot condensation of an aldehyde with alkyl acetoacetate and ammonia was developed more than hundred years ago. In the forties the interest for this substance class increased due to their pharmacological activity.[2] 4-Aryl-1,4-dihydropyrdines form an important class of calcium channel antagonists.[3]

When sterically hindered aldehydes are employed in classical Hantzsch synthesis, long hours of reflux are needed, but still the yields are generally low.[4,5] Reactions assisted with microwave dielectric heating usually gives shorter reaction times and often higher yeilds compared to conventional methods[6] and has lately become a popular method. In order to evaluate the possibility to increase the yield we used a Smith Synthesizer™ from Personal Chemistry, a mono-mode microwave synthesizer with both temperature and pressure control.

With the use of Smith Synthesizer we here show that Coherent Synthesis are a rapid method compared to conventional heating for producing high yielded library synthesis of 1,4-dihydropyridines. We also shown that due to the temperature control provided by the Smith Synthesizer™, time and temperature could be varied independently of each other, which made it possible to increase the yield for this typical substance class compared with both conventional methods and the use of domestic microwave ovens.

1 Hantzsch, A., *Justus Liebigs Ann. Chem*, **215**, 1882, 1–82.
2 Goldmann, S., Stoltefuss, J., *Angew. Chem. Int. Ed. Engl.*, **30**, 1991, 1559–1578.
3 Di Stilo, A., Visentin, S., Clara, C., Gasco, A. M., Ermondi, G., Gasco, A., *J. Med. Chem*, **41**, 1998, 5393–5401.
4 Loev, B., Goodman, M. M., Snader, K. M.,Tedeschi, R., Macko, E. J., *J. Med. Chem*, **19**, 1974, 956–965.
5 Kuthan, J., Palecek, K., *Collection Czechoslov. Chem. Comm.*, **39**, 1974, 3711.
6 Majetich, G., Hicks, R., *Res. Chem. Intermed.*, **20**(1), 1994, 61–77., Strauss, C. R.,Trainor, R.W., *Aust. J. Chem.*, **48**, 1995, 1665–1692., Caddick, S., *Tetrahedron,*, **36**(11), 1995, 10403–10432.

### A new concept for the on-line storage of sample plates, integrated with plate preparation for high-throughput screening and 'cherrypicking'

*Jacques H. M. van den Broek, Will H. A. Kuijpers and Thijs de Boer, NV Organon, Oss, The Netherlands; and Jonathan H. Connick, Organon Laboratories, Newhouse, UK*

Recently Organon installed automated screening and plate preparation systems for its research facilities in Oss (The Netherlands) and Newhouse (UK). These robotic systems have been developed in close collaboration between Organon and Scitec Laboratory Automation (Lausanne, Switzerland).

Each of the systems consists of three linear track robots, one of which performs the screening process using standard peripherals. The other two robots take care of the plate preparation and 'cherrypicking' procedures. To this end, copies of our total mother plate collection are stored under controlled conditions in Scitec plate stackers (AutoStack) that can be addressed by one of the two robots. The system is designed in such way that the loading and refreshment of the on-line storage, screening-plate preparation, and ‘cherrypicking’ can be executed automatically in 24 hours operation.

A more detailed design of the system and the rationale behind it will be further disclosed.

### Evaluating the performance and capacity of an MTP and microtube Storage Facility

*Thorsten Pötter*[*,#], *Frank Böhme*[*], *Olaf Brackhagen*[*], *Frank Marciniak*[*], *Wolfgang Röben*[*], *Hermann Stein*[*] *and Otto Ramsebner*[†]

By deciding to extent the screening cascade in the agriculture business unit of Bayer AG by implementing High Throughput Screening we were confronted by many questions:

- How will we screen in HTS mode?
  — single compounds vs. mixture
  — plate formats
  — throughput
- How to validate first screening information?
  — retest
  — dose response information
  — other tests
- How to store the compounds?
- liquid vs. solid
- frozen or not
- How to provide compounds?
  — ready to use or not
  — volume and concentration

In answering these questions it became obvious that we had to design a new storage system to support two new HTS-robots and several workstations. We decided to screen single compounds in a 384 plate format and to validate HTS-hits by dose response information. Compounds should be provided more or less ready to screen as DMSO solutions, therefore several copies must be stored in MTPs and Microtubes (384 tubes on a rack with MTP footprint). For quality reasons these solutions should be frozen and the number of freeze/thaw cycles should be limited. These assumptions, the expected number of tests per year and the size of the library were used to estimate the capacity and the desired throughput of the storage system.

Meanwhile a REMP system with capacity of more than 130,000 storage positions is in place and we have been able to measure exact movement times of the robot and access times to the MTPs and Microtubes.

Depending on our assumptions the throughput varies, especially for the Microtubes. With the same equipment the output of tubes per hour ranges from 88 tubes/hr to more than 1000 tubes/hr. The input/output of MTPs varies only from 91 to 162 MTPs/hr. The poster shows in detail how these results were compiled.

* Bayer A G, Crop Protection Business Group, D-51368 Leverkusen, Germany.

† Remp A G, Laboratory Automation, Oberdiessbach, Switzerland.

### Automated flow system for the reformatting and dispatch of screening samples in duplicate vials

*David Jacobs, Oxford Asymmetry Int., Oxford, UK*

Oxford Asymmetry International is a provider of sophisticated chemical services to the pharmaceutical, agrochemical and biotechnology industries. The Discovery division prepares multiple compound libraries for both lead discovery and lead optimisation. These compounds are reformatted prior to dispatch so they can be seamlessly incorporated into our clients’ compound archives, requiring different reformatting procedures to be used for each client.

The collaboration in question required the synthesis, analysis, reformatting and dispatch of 200,000 compounds over a 2-year term. Each sample was transferred from microtitre plates into duplicate vials and the weight of material in each vial accurately determined.

This poster will highlight the processes involved in the reformatting of these ~200 K screening samples using the Zymark XP weigh station and Gilson 215 liquid handlers. It describes the handling and transfers of samples and indicates the amount of data generated both electronically and in hard-copy format.

This presentation will demonstrate the direct impact of laboratory automation in delivering large numbers of combinatorial samples.

The whole process routinely dealt with ~9 K compounds/month and this poster will highlight the role the Zymark XP robot weigh stations played.

### Use SciClone with robotic system to perform multiple assays and assay optimization

*Randy Yen, John Hanson and Wai Lee Wong, Assay & Automation Technology Department, Genentech, Inc., South San Francisco, CA*

We have set up a few ORCA and CRS robotic systems to handle the high throughput screening assay, but there are still a lot of regular assays needs to be run by analyst manually, because those are not for high throughput screening, so the samples amount are low. It is not good to put on the robotic system. To automate those assays efficiently, we decide to modify our robotic system with SciClone station to handle those assays at the same run by continuing using Clara LT scheduling software and without changing the current hardware setting.

The Clara LT scheduling software usually handle one assay at a time, but by using SciClone station, we are able to run multiple assays at the same time. We have taken an advantage of SciClone ActiveX communication to develop our own DDE SciClone driver to handle the activity between the robot system and SciClone station. It gives us the flexibility to use SciClone with the ORCA robotic system efficiently. Beside enhance the communication for the system, we have to improve the sample tracking method for our robotic system, so we are able to handle multiple assays in one robotic system and use the robot as a assay develop tool to for the assay development.

a. To handle multiple assays: First, we need to identify those assays have the same incubation time and development time and then batch them together in one run, so Clara LT will treat all of them as one assay. Second, we need to program the robotic control software to track the sample number and identify which reagent should be used for each sample then the program will instruct the SciClone station to treat them as a different assay and pipette proper reagent to each plate. By this way, analyst can combine several different assays run in one robotic run. The robotic system will run all of them at once without trouble.

b. To optimize an assay: We use all the position on the SciClone deck to setup several different reagents and leave one position for tips box. By using our new program, robot can instruct SciClone to pipette proper reagent to the associated sample plate. In one run. Analyst can test several different reagents at once. It can be used to optimize the assay quickly and speed up the assay development for scientist.

### Automated antibiotic susceptibility assays

*Ken Coleman[1], Randall Engler[2], Stewart Pearson[1] and Eudell Swann[1], [1]SmithKline Beecham Pharmaceutical Research, Collegeville, PA [2]Kendro Laboratory Preoducts, Newtown, CT*

Standard antimicrobial susceptibility testing typically requires 18–24 h incubation of organism in a range of antimicrobial concentrations before a growth reading is taken and used to determine the Minimum Inhibitory Concentration (MIC) of the drug.

For high-throughput assays, where large numbers of compounds are screened and few are expected to be active, a single, high concentration of agent can be used and hits then progressed to a full MIC assay, with a saving of time, labour and compound. The sensitivity of this assay can be increased by taking readings at intermediate times, such as 8 & 16 h, making it possible to identify (1) Agents whose MIC is 2–8× higher than the working concentration (2) Active agents which are breaking down over time.

Both types of assay have been successfully automated with the integration of an Heraeus *cytomat*™ 6000 incubator (Kendro, Newtown, CT), two Microlab AT2 Plus plate processors (Hamilton, NV), a Multiscan Ascent plate reader (Labsystems, MA) and an R16 tracked robot (ST Robotics, NJ), using the Overlord procedural language (Process Analysis and Automation, UK).

### Just-in-time compound delivery to multiple kinase targets for concurrent specificity screening

*Brent Butler, Cole Harris, Stephanie Schweiker and Edgar Wood, Molecular Biochemistry Department, Glaxo Wellcome Inc. Research Triangle Park, NC*

We have developed a simplified method for serially diluting and delivering compounds to multiple sets of assay plates. This has permitted us to concurrently screen large numbers of compounds against multiple kinase targets in a just-in-time manner. The data generated in these screens allows us to quickly and reliably determine the potency and specificity of kinase inhibitors.

### Use of laboratory automation in the drug discovery groups

*Joseph A. Short, Thomas L. Lloyd and Eric D. Lynch, DuPont Pharmaceuticals Company, Newark, DE*

The use of laboratory automation in our discovery group has drastically increased the throughput of test compounds. Of the different kinds of lab automation in our department, our discovery group uses three types: The Zymark PyTechnology robot, Packard MultiProbe II and Tomtec Quadra96 SPE Workstation. The Zymark robot was one of the first pieces of lab automation used in our group. It is used for completing serial dilutions for standard and quality control samples. It is also used for solid supported liquid/liquid extractions (Chem Elut) and on-line protein precipitation extractions. The Packard MultiProbe and Tomtec Quadra96 are liquid handlers, used primarily for solid phase extraction using 96 well microplates. The use of these tools in our drug discovery group will be presented and discussed.

### Fully automated anti-varicella Zoster enzyme-linked immunosorbent assays supporting manufacture of a novel intravenous therapeutic for treatment of aliments caused by the Varicella Zoster Virus

*LeeAnne Macaulay, Andrea Masi, Lori Soluk and Danuta Kierek-Jaszczuk, Cangene Corporation, Winnipeg, Manitoba, Canada*

A novel liquid-based therapeutic VZIG has recently been developed at Cangene, which specializes in the manufacture of human plasma-based pharmaceuticals. The manufacture of this pharmaceutical is supported by two VZV ELISAs, a commercial Wampole Laboratories VZV IgG ELISA® and an in-house developed, amplified antibody-capture ELISA, which were validated for the quantitation of the Varicella Zoster Virus specific antibodies in the finished product and patient sera, respectively.

The purose of the present studies was to impove and streamline the performance of the assays by automating several procedural steps that are of a crucial importance for the ELISA performance. The AT*plus* 2 Liquid Handler, working in tandem with the Microlab F.A.M.E. Plate Analyzer were used to automate: (i) the preparation and pipetting of serial dilutions in 96-well microtitre plates, and (ii) downstream processing of the plates. The plate-processing steps included incubation, plate washing, reagent addition, photometric reading as well as data capturing and management. All liquid handling and plate-processing steps were pre-programmed using the software of the respective Microlab instrument. The designed and programmed protocols closely followed the procedures detailed in the Standard Test Methods for the validated commercial Kit and in-house ELISA and incorporated the steps allowing for a positive identification of the processed microplates.

The automated ELISAs were performed as single or multiple plate experiments. After serially diluted samples were automatically prepared and pipetted into 96 wells of the microtitre plates, the plates were inserted into the F.A.M.E. for further processing in accordance to the programmed protocols. On the conclusion of the experiment, a printout with the raw and blank reduced data was obtained and the data was graphed and analyzed using the Excel spreadsheet program. It has been shown that both automated ELISAs were capable of accurate and reliable quantitation of the anti-VZV antibodies from standardized preparations. This proves that both VZV ELISAs can successfully be performed, in a fully automated manner, on the platform offered by the Microlab instruments. The automated ELISAs offer an advantage of the increased data security and traceability as well as reduction of human error.

### Automated plasma protein binding screen: implementation on a Tecan Genesis and Zymark RapidPlate®

*Kelly Jordan, Jimmy Bruner, Cosette Serabjit-Singh and Stephen A. Wring, Glaxo Wellcome Inc., Research Triangle Park, NC*

Drug binding to plasma proteins can markedly affect disposition, and early information on binding could afford more rapid drug design and selection. Unfortunately, established methods for determining plasma binding are not amenable to drug discovery as they are laborious and not readily automated.

To improve throughput we have developed an automated method employing a novel 96 well ultrafiltration format, with a Zymark RapidPlate® and a Tecan Genesis 150, that increases capacity 4 fold to 32 compounds per run.

The RapidPlate® prepares analytical standards in drug-free plasma and ultrafiltrate, and also spikes test plasma. After incubation, the Genesis transfers the incubated plasma samples to a Millipore Microcon ultrafiltration plate that is centrifuged to produce ultrafiltrate. The Genesis then collects the ultrafiltrate and the Glaxo Wellcome Balance Data Collection system determines the volume produced. The Genesis uses the volume data to restore plasma retentate back to its starting volume by addition of drug-free ultrafiltrate. Drug concentrations are determined in ultrafiltrate and plasma retentate samples, thereby allowing calculation of the bound fraction (Fb) and mass balance.

The method has been applied to an evaluation set of compounds and data show good agreement with literature values of Fb over the range of 10–99.5%.

### High-throughput screening based on multiplexed CE and absorption detection

*Edward S. Yeung, Xiaoyi Gong, Lianjia Ma, Seong Ho Kang and Yonghua Zhang, Ames Laboratory-USDOE and Department of Chemistry, Iowa State University, Ames, IA*

Capillary electrophoresis (CE) is now a mature technique for analytical separations. In its various modes, impressive performance has been demonstrated for ionic as well as for neutral compounds. The Human Genome Project provided the impetus for developing multiple capillary systems. However, capillary arrays are not just for DNA analysis. Every CE protocol can be similarly multiplexed to achieve higher throughput and reduced reagent consumption without sacrificing its good resolving power and full automation. To extend the applicability to non-fluorescing compounds, we constructed a simultaneous multiplexed absorption detector for 96 capillaries. Entire sample trays in the 96-well microtiter plate format can be analyzed in one operation. This high-throughput capability gives CE a unique advantage over column liquid chromatography in most applications. Examples from DNA analysis, enzyme assay, peptide mapping and combinatorial synthesis will be presented.

### The determination of membrane affinity using solid supported lipid bilayers (Transil®)-lipophilicity in high-throughput processes

*T. Hartmann, M. Schöttner and A. Loidl-Stahlhofen, NIMBUS Biotechnology GmbH, Leipzig, Germany*

Quantification of lipid binding performance of pharmaceuticals is very important in pharmacology, medicine and biochemistry. The huge amount of new compounds generated by combinatorial chemistry requires a quick and automated lipophilicity assessment. In case of pharmaceuticals the octanol-water partition coefficient o/w is a popular descriptor of the pharmacokinetic behavior in biological systems. Nevertheless, whenever surface active and/or charged compounds are to be investigated, the Po/w is prone to fail as the bulk phase octanol is unable to mimic the anisotropic membrane behavior. Therefore membrane model systems are often applied for quantifying membrane affinity. Usually, they achieve a better correlation, but show severe drawbacks especially due to the enormous expense of time needed for data acquisition. We develop a new process for the high-thoughput-screening of lipid/water partition coefficients (Plipid/water) to overcome the limitations of Po/w: Solid supported lipid bilayers (TRANSIL®) combined with HPLC-technique allow a fast, easy and reproducible determination of membrane affinity (lipophilicity).

### TRANSIL®, a new tool for functional immobilization of transmembrane proteins by lipid bilayers on a solid support for high-throughput processes

*J. Noeller, J. Schmitt and S. Kaufmann, NIMBUS Biotechnology GmbH, Karl-Heine Straße 99, D-04229 Leipzig, Germany*

Rational design of new drugs requires a fast and reliable access to their interaction properties with cellular membranes such as (i) binding to the target proteins (ii) drug targeting, membrane affinity, and permeation. Here, solid supported lipid bilayers—with or without immobilized membrane proteins—are of key interest in the fields of pharmaceutical screening, biosensors and bioseparation. Their major advantage is improved long term stability and greater ease of handling as compared to systems without support i.e. vesicles, cells or proteoliposomes. TRANSIL®, a new surface consisting of supported lipid bilayers, presents an easy access to the investigation of transmembrane proteins (like GPCR-proteins) in the fields of pharmaceutical screening and biosensors. To mimic cellular membranes, TRANSIL® is optimized in regard to lipid composition and bilayer-support interactions. Therefore, transmembrane proteins are readily immobilized in a matrix similar to their natural environment, showing no significant loss of activity and even preferred orientation. In combination with state-of-the-art detection tools, this versatile system is very suitable for the assessment of ligand binding, protein-protein interactions and general functional studies of receptors and transporters or other transmembrane proteins.

**Fast synthesis at the Janssen research foundation**

*Davy Petit, Marc Schroven, Janssen Research Foundation, Beerse, Belgium*

This poster gives an overview of how parallel synthesis is performed in the Fast Synthesis Lab, a subunit of the combichem group at the department of medicinal chemistry. The entire workflow and dataflow are automated. A Zymark robot is used for lab-intensive manipulations. An automated preparative LC/MS purification system delivers compounds with a minimum purity of 90% at an average yield of 50 mg. A high throughput analysis tool determines the purity of the compounds based on MS and UV data. Data handling (calculations, registration,...) is accomplished using MS Excel, Accord Combichem and Accord for Excel.

**An electrochemiluminescence-based assay for cAMP**

*Tamyra Shafer, Jim Schmidt, James W. Karaszkiewicz and Elizabeth Kenten, IGEN International, Inc., Gaithersburg, MD*

Cyclic adenosine monophosphate (cAMP) is generated by cells in response to the activation of certain G-Protein coupled (GPC) receptors. Increases in the levels of intracellular cAMP affect a wide range of cellular processes. The measurement of cellular cAMP levels is an important tool in basic studies of G-protein coupled receptors, and screens for compounds that affect GPC-receptor function. We describe an ORIGEN® assay that measures intracellular cAMP using a competitive immunoassay format. The procedure combines the lysis of the cellular samples and the competitive binding reactions into a single incubation step; reagents may be added directly to cells in microwell plates.

Products of the immunoassay binding reactions are quantitated by electrochemiluminescence detection using ORIGEN® instrumentation such as the M-SeriesTM M-8 Analyzer. The cAMP assay has a detection limit of less than 10 pmol/ml and a wide dynamic range extending to concentrations greater than 1,500 pmol/ml. The protocol is simple, does not require sample pretreatment or wash steps and lends itself well to automation. Acetylation of cAMP is not required. The ORIGEN cAMP assay illustrates the usefulness of electrochemiluminescence detection for the measurement of analytes in complex matrices such as cell lysates.

**Corning 1536-well assay plate for HTS**

*Arthur Trombley, Corning Inc. Science Products Division, Portsmouth, NH; Jill Veilleux, Corning Inc. Science Products Division, Action, MA; David Dunn and Marc Orlowski, Pharmacopeia, Inc., Princeton, NJ; Meng Zhang and Doug Boyd, LJL Biosystems, Inc., Sunnyvale, CA*

The new Corning 1536-well 2 µl Plate provides the foundation for a unique solution to assay miniaturization for high throughput screening. The advantages of the 1536-well HTS plate include:

(1) Conservation of 96 and 384 well format, which aids in sample transfer, well identification, and data manipulation.
(2) Low profile, which provides increased sample density over conventional plate height and reduces parallax effects in imaging systems.
(3) Three point positioning and tight manufacturing tolerances for all dimensions, which enable high-speed automated plate handling functions.
(4) The lowest volume wells (2 µl) that are commercially available.
(5) Optimized materials for manufacture, which enhance performance in fluorescence, luminescence and colorimetric detection formats.

Here, we probvide an analysis of evaporative loss, automated dispensing at low volumes, coupled to detection of luminescent substrate. The Corning 1536-well 2 µl Plate is shown to be an ideal solution to scale up from traditional 96 and 384 well formats in high throughput screening applications.

**Fluorescent cell based assays for high throughput analysis**

*Jeanne Phillips and Aldo Pitt, Millipore Corporation, Danvers, MA*

Ideally suited for high throughput applications, cell based fluorescent and time-resolved fluorescence (TRF) assays are typically less expensive and hazardous than radioactive assays and are versatile, sensitive, and quantitative. Common uses include detection of cell-cell adhesion, cell viability, proliferation, cell cycle determination, apoptosis as well as detection of specific proteins/receptors.

In our present study, we determined the incorporation of a fluorescent probe precursor (calcein AM) by measuring intracellular fluorescence both in suspension and in adherent cell lines. Calcein AM is cleaved by intracellular esterases to form calcein, a pH-independent, cytosolic fluorescent marker. Calcein AM uptake was measured in cyclosporin or verapamil inhibited P-glycoprotein (Pgp expressing MDCK and MES-SA/MX2) cells. The MultiScreen®-PCF and FL plates possess some highly desirable features for automated high-throughput cell based fluorescent screening procedures. In addition to culturing the cells, all the subsequent assay steps (such as media exchanges, drug treatment, washing, and reading) can also be performed directly in the same MultiScreen plate with a significant reduction in process time.

### Performance validation of an automated high throughput plasmid purification system

*Michael J. Domanico, Jamie L. Noble, Natalie Y. Trees, Joseph A. Hensley, Preston B. Hradecky, Kelly M. Clark and Randolph J. Hellwig, Eppendorf-5 Prime, Inc., Boulder, CO*

The PERFECTprep automated plasmid purification system proved to be very reliable and robust both in internal and external validation studies. The system was rigorously tested internally by performing 4800 purifications per day for two weeks. As part of the platform installation process, 9600 purifications were also performed at each of three genome sequencing centers. A total of six host/vector combinations were utilized to demonstrate product reliability and consistency within different cell types.

Agarose gel analysis revealed excellent reproducibility and uniformity of the purified plasmid, with yields of 100 ng/μL in more than 90% of the preps. The DNA sequencing quality averaged 475 base pairs of PHRED Q20 for the entire validation study. These results conclusively demonstrate that this plasmid purification system is robust and capable of producing sequencing quality plasmid in a high throughput mode.

